# Coherent Somatic Mutation in Autoimmune Disease

**DOI:** 10.1371/journal.pone.0101093

**Published:** 2014-07-02

**Authors:** Kenneth Andrew Ross

**Affiliations:** Department of Computer Science, Columbia University, New York, New York, United States of America; Pavillon Kirmisson, France

## Abstract

**Background:**

Many aspects of autoimmune disease are not well understood, including the specificities of autoimmune targets, and patterns of co-morbidity and cross-heritability across diseases. Prior work has provided evidence that somatic mutation caused by gene conversion and deletion at segmentally duplicated loci is relevant to several diseases. Simple tandem repeat (STR) sequence is highly mutable, both somatically and in the germ-line, and somatic STR mutations are observed under inflammation.

**Results:**

Protein-coding genes spanning STRs having markers of mutability, including germ-line variability, high total length, repeat count and/or repeat similarity, are evaluated in the context of autoimmunity. For the initiation of autoimmune disease, antigens whose autoantibodies are the first observed in a disease, termed primary autoantigens, are informative. Three primary autoantigens, thyroid peroxidase (*TPO*), phogrin (*PTPRN2*) and filaggrin (*FLG*), include STRs that are among the eleven longest STRs spanned by protein-coding genes. This association of primary autoantigens with long STR sequence is highly significant (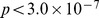). Long STRs occur within twenty genes that are associated with sixteen common autoimmune diseases and atherosclerosis. The repeat within the TTC34 gene is an outlier in terms of length and a link with systemic lupus erythematosus is proposed.

**Conclusions:**

The results support the hypothesis that many autoimmune diseases are triggered by immune responses to proteins whose DNA sequence mutates somatically in a coherent, consistent fashion. Other autoimmune diseases may be caused by coherent somatic mutations in immune cells. The coherent somatic mutation hypothesis has the potential to be a comprehensive explanation for the initiation of many autoimmune diseases.

## Introduction

I have previously provided evidence that somatic gene conversion and/or deletion in sequence harboring long segmental duplications is correlated with disease [1]. According to this hypothesis, autoimmunity is a response to novel (somatically mutated) antigens. Others have proposed a role for somatic mutation in autoimmunity [2], [3]. The remarkable extent of somatic mutation, including copy number variation and somatic mosaicism, has recently been elucidated, with several proposed links to neurological disease [4]–[9]. The connection between somatic mutation and autoimmunity requires that somatic mutations be coherent [1], i.e., that the same type of mutation occur in many cells, to the point that the somatically mutated protein either disrupts normal function or is noticed by the immune system as non-self. A coherent mutation may be recurrent (occuring independently in many cells) [10] or clonal (occuring once and replicating many times).

### Somatic Mutation of Tandem Repeat Sequence

Coherent somatic mutation of the haptoglobin gene (*HP*) has been observed in vivo in humans [11]. Carriers of the *HP2* allele have a segmentally duplicated 1.7kb sequence fragment within the gene that includes two additional exons beyond the shorter *HP1* allele. In an *HP2* homozygote, Asakawa et al [11]. found a shorter DNA sequence corresponding to an exact excision of one copy of the tandem repeat. In each of several *HP2* homozygotes subsequently tested, a small but measurable concentration of the shorter sequence was identified. Asakawa et al. argued that rare but regular somatic deletion events occur in vivo. In the mouse, a similar kind of somatic mutation has been observed in vivo at a longer 70 kb segmental duplication [12], [13]. The mutation frequency was much higher than for *HP* in humans, presumably due to both the longer duplicon and the fact that phenotypic measurement was performed in gene-expressing tissues where mutations would be more common, rather than in blood cells [11], [14]. Somatic mutation at additional loci, mediated by inverted repeats [15] or tandem repeats [16], has been observed in vivo in humans.

Long segmental duplications are not the only repetitive sequence subject to high mutation frequencies. Simple tandem repeats (STRs), including microsatellites and minisatellites that are highly mutable in germ-line cells, are also mutable in somatic cells [17], [18]. Some STRs encode proteins, and somatic mutations would generate novel, potentially immunogenic proteins. While not strictly an STR, such an effect has been observed at the La antigen associated with Systemic Lupus Erythematosus (SLE) and Sjogren's Syndrome (SJ), where somatic mutations of an 8bp poly-A sequence into a 7 bp mutant have been observed [19]. These mutations correlate with autoimmunity, in that about 30% of La-reactive SLE/SJ patients respond specifically to the mutant protein [19] and somatic mutant DNA can be detected in such individuals [20].

Other STRs occur within introns, where changes in repeat counts can change splicing behavior [21]. Altered splicing of autoantigens has been proposed as a mechanism for generating immunogenic protein variants [22]. In particular, inflammation can lead to reduced levels of the splicing factor ASF/SF2 [22]. Low levels of ASF/SF2 are associated with DNA double strand breaks and DNA rearrangements triggered by R loops between DNA and transcribed RNA [23]. R loops promote instability in GC-rich trinucleotide repeats [24], suggesting that transcribed repetitive sequence may be particularly vulnerable to somatic mutation induced by ASF/SF2 depletion.

Additionally, repeat mutations are often accompanied by significant changes in methylation [25]. Demethylation can potentially lead to aberrant transcription initiation in the middle of the gene sequence [26]. Repetitive sequence is also an essential factor in cellular mechanisms for methylating nearby sequence [27], [28]. Changes to the methylation pattern can also affect splicing [29]. Altered methylation patterns have been observed in several autoimmune diseases [30].

Yet another reason to focus on somatic repeat mutations in autoimmune disease is the observation that somatic tandem repeat mutations can be induced by inflammation typical of an immune or autoimmune response [31], [32]. This observation provides the basis for a feedback loop. An initial immune response against a pathogen could, as a side-effect of inflammation, trigger the initial production of aberrant protein. The aberrant protein induces a second immune response, with further inflammation and coherent somatic mutation in nearby cells (or remote cells opsonized by autoantibodies [33], [34]) creating a cycle of autoimmunity. Anti-inflammatory medications reduce rates of somatic mutation in some cancers [35], further supporting a link between inflammation and somatic mutation,

Human STR sequence is overabundant near telomeres [18], [36]. Nevertheless, the germ-line variability of a minisatellite repeat in a population does not depend on its chromosomal location [37]. Instead, the primary determinants of minisatellite variability are (a) the number of repeat units it contains, and (b) the degree of identity between different repeat units within the sequence [37]. Variability is a nonlinear function of these measures: Doubling the copy number increases the probability of being variable about 15-fold, and adding 10% to the repeat unit similarity increases the probability of being variable about 18-fold [37]. A more recent model also takes into account the size of the repeat unit [38]. The total repeat length (i.e., the product of the repeat unit size and the repeat count) is strongly correlated with variability [38]. For segmental duplications, high sequence identity is most important for structural variability, with high duplicon length and low duplicon separation also playing a role [39].

While somatic and germ-line microsatellite mutation patterns appear similar [18], somatic and germ-line mutation patterns differ for minisatellites [40]. Germ-line minisatellite mutations involve recombination-based repair of double strand breaks (DSBs), while sponteneous somatic minisatellite mutations arise by replication slippage or mitotic recombination [40]. For somatic mutations induced by inflammation [31], [32], DNA damage appears to be critical, including DNA strand breaks [41]. The resulting mutation patterns in STRs may therefore more closely resemble germ-line mutations or somatic mutations in cancer [42] than spontaneous somatic mutations. Structural mutations in repetitive sequence are orders of magnitude more frequent than point mutations [43]. Mitotic mutation rates of up to 2% have been observed in the longest human tandem repeat sequences [44].

### Autoimmunity

Autoimmune diseases have overlapping features, including shared susceptibility loci [45]–[48] and cross-heritability [49]. Nevertheless, each autoimmune disease has specific manifestations, causing damage to particular organs or systems. The central enigma of autoimmune disease is why a relatively small set of specific proteins are immunologically targeted [50]. Many, but not all autoantigens in systemic autoimmune diseases are proteins that are cleaved during apoptosis [51], [52], but the reason for this association is unclear given that T cell tolerization to such cleaved proteins is expected [52], [53]. Autoantigens appear to have longer exons and harbor more SNPs than other genes [3], [54], and they are enriched in several biologically relevant categories [3].

The most prominent phenotype of autoimmune disease is the presence of specific antibodies (Tables 1 and 2). While T-cell epitopes are also implicated in autoimmunity, they are more difficult to measure [55]. Mutant protein can induce antibodies to wild-type protein, even when T-cell tolerance to wild-type protein is maintained [56]. Thus, antibodies are likely to provide the most robust signal about autoimmune targets.

**Table 1:** Twenty-one of the most prevalent human autoimmune diseases, in approximately decreasing order of prevalence [49], [275].

Abbrev.	Name	*PTPN22* Assn.	B-cell Autoantigens	Refs.
GD	Graves' Disease	Yes [45]	**TPO**, **TG**, TSHR	[276]
RA	Rheumatoid Arthritis	Yes [143]	**FLG**, **VIM**, **FGA**, **FGB**, **ENO1**, IgG (rheumatoid factor), IFI16, ANXA1, PADI4	[169], [178], [277]–[282]
HT	Hashimoto's Thyroiditis	Yes [143]	**TPO**, **TG**	[276], [283]
CEL	Celiac disease	Unclear [284]–[286]	TG2, HP, actin, CALR, TG3, ganglioside, collagen	[57], [287], [288]
PSO	Psoriasis	No [143]	PALLD, AGAP3, DSP, collagen-21, ATXN3	[289]
VIT	Vitiligo	Yes [290]	TYR, TH, TYRP1, MCHR1, lamin A	[291]–[293]
SJ	Sjogren syndrome	Unclear [294], [295]	**SPTAN1**, SPTBN1, Ro52(TRIM21), Ro60(TROVE2), La(SSB), CHRNA3, IFI16, VIM, CHRM3	[178], [296]–[302]
UC	Ulcerative Colitis	Yes [105]	**HMGB1**, **HMGB2**, **pANCA**, tropomyosin	[303]–[305]
AS	Ankylosing Spondylitis	No [306]	multispecific	[307]
T1D	Type-1 diabetes	Yes [143]	**PTPRN2**, **PTPRN**, INS, GAD2, SLC30A8, VAMP2, NPY; AMY2A (fulminant T1D)	[308]–[313]
AA	Alopecia Areata	Yes [314], [315]	TH, TCHH, KRT16	[316]–[318]
JIA	Juvenile Idiopathic Arthritis	Yes [319], [320]	DEK, HSP70, citrullinated peptides	[321]–[324]
PA	Pernicious Anemia	Unknown	ATP4A/ATP4B, pepsinogen A	[325]–[327]
MS	Multiple Sclerosis	No [143]	**MAG ** 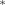 , MBP, PLP, MOG, CRYAB, CR1, neuronal antigens	[150], [151], [328]–[332]
CD	Crohn's disease	Opposite 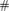 [333]	GP2, CUDZ1	[334]
SLE	Systemic Lupus Erythematosus	Yes [143]	**Ro60(TROVE2)**, **SNRPA**, **APOH/cardiolipin-complex**, **ribosomal P**, VIM/cardiolipin-complex, La(SSB), Ro52(TRIM21), ds-DNA, Sm, SNRNP70, SNRPC, chromatin/histones, Ku, CALR, NCL, RF, CR1, IFI16, VIM, lamin B, F2, F2/Phosphatidylserine, ANXA1, ANXA2, ANXA5, NPM1, HMGB1, LTF, SR proteins, others	[178], [281], [299], [328], [335]–[352]
UV	Uveitis	No [353]	CRALBP, CRYAA, CRYAB, CRYBB1	[354], [355]
AD	Addison's disease	Yes [356]	**CYP21A2**	[357], [358]
MG	Myasthenia Gravis	Yes 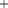 [359]–[362]	AChR, MUSK, LRP4, AGRN, ColQ, TTN, KCNA1, RYR	[363]–[365]
DM	Dermatomyositis	Yes [366]	Mi-2-complex, IFIH1, TRIM33, MORC3, Ro52(TRIM21)	[367], [368]
SSc	Systemic Sclerosis	Yes [369], [370]	RNA Polymerase III, CENPB, CENPA, RNA Polymerase I, RNA Polymerase II, TOP1, PM/Scl-complex, Ro52(TRIM21), SNRNP70, NOR-90, Ku, Th/To, U3RNP/FBL, IFI16, ANXA5, NPM1, HMGB1, HMGB2, Mitochondrial-M2	[211], [281], [299], [371]–[375]

Autoantibodies to antigens in bold are known to be primary antibodies that occur early in disease progression, often prior to the appearance of symptoms. The tryptophan allele of the Arg620Trp polymorphism at rs2476601 in the *PTPN22* gene is associated with many autoimmune diseases, as indicated in the “*PTPN22* Assn.” column. Atherosclerosis (CAD) is not universally considered an autoimmune disease, and is therefore not listed. Nevertheless, CAD does have autoimmune features [376] and an association with *PTPN22* [377]–[379]. 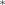
The initial pathology in some MS lesions is associated with MAG loss [329], [380], [381].

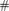
The tryptophan PTPN22 allele is protective from CD [333].

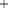
In MG, two studies conflict about whether PTPN22 is specifically associated with the subset of cases having anti-TTN antibodies.

**Table 2:** Autoantigens for selected low-prevalence autoimmune diseases.

Abbrev.	Name	Autoantigens	Refs.
PV	Pemphigus Vulgaris	DSG3, DSG1, HLA-DRA, DSC3, DSC1, ATP2C1, PKP3, CHRM3, COL21A1, ANXA8L1, CD88, CHRNE	[382], [383]
RHF	Rheumatic Fever	VIM, MYBPC3, tropomyosin, collagen	[186], [384]
LEMS	Lambert-Eaton Myasthenic Syndrome	CACNA1A, CACNB2	[385], [386]
AH1	Autoimmune Hepatitis (type 1)	HMGB1, HMGB2	[387]
AH2	Autoimmune Hepatitis (type 2)	CYP2D6, CES1, PDIA3	[388], [389]
HA	Autoimmune hemolytic anemia	RHD, GYPA	[390]
AP	Autoimmune pancreatitis	AMY2A, CA2, LTF, HSP10, plasminogen-binding protein, trypsinogens, SPINK1	[313], [391]
PBC	Primary Biliary Cirrhosis	Mitochondrial-M2, SP100, PML, NUP210, Ro52(TRIM21), CENPB, SUMO2, SUMO1, CHRM3	[210], [392], [393]
NMO	Neuromyelitis Optica	AQP4	[329]
GPS	Goodpasture's Syndrome	COL4A3	[394]

A B cell epitope does not have to be from the same protein molecule as the T cell epitope in order for the B cell to be activated by a CD4+ (helper) T cell. A B cell that endocytoses a protein complex by binding to one of its proteins can be activated by a CD4+ T cell specific to another protein in the complex. Such a mechanism has been used to explain anti-TG2 antibodies in celiac disease, where a TG2-specific B cell is activated by a CD4+ T cell specific to gliadin after endocytosis of a TG2-gliadin complex [57].

Thus, a protein is a candidate CD4+ T cell target either if it elicits antibodies itself, or if an in-vivo binding partner of the protein elicits antibodies. B cell specificities (and thus antibodies) to multiple proteins can be supported by a single CD4+ T cell epitope. I use the term *peri-antigen* to mean an in-vivo binding partner of an autoantigen. A peri-antigen can potentially function as a CD4+ T-cell target supporting B cell specificity to the autoantigen.

### Testing the Coherent Somatic Mutation Hypothesis

I sought data to test the hypothesis that autoimmune disease is associated with mutable repetitive sequence. Because of its construction from long contigs [58], the reference human genome has reliable sequence for most repetitive regions, although gaps still remain. Because shorter reads were used, the Celera sequence is missing the interiors of many repetitive elements [59]. Most current sequencing technologies use short reads that must be assembled into whole genomes. Both de-novo assembly and alignment-based assembly are unreliable in highly repetitive regions [60]–[62]. The reference human genome is therefore the primary currently available source of robust repetitive sequence throughout the genome.

Antibodies that develop early in disease progression provide the strongest evidence for a causative role for the corresponding antigen. A *primary* autoantigen is one whose antibodies have been shown, in at least a subset of cases, to be the first disease-associated antibodies to appear. A test of the coherent somatic mutation hypothesis can be formulated as follows: Is there a statistical link between primary autoantigens (and/or their peri-antigens) and genes containing highly mutable sequence?

Once such a statistical link is established, a subsequent test of the comprehensiveness of the coherent somatic mutation hypothesis would consider other mutable (e.g., long STR) sequence. To what extent could somatic mutation at these loci explain other autoimmune phenomena?

## Results

### Genes Containing Long Repeats Include Primary Autoantigens for Common Autoimmune Diseases

**Figure 1:**
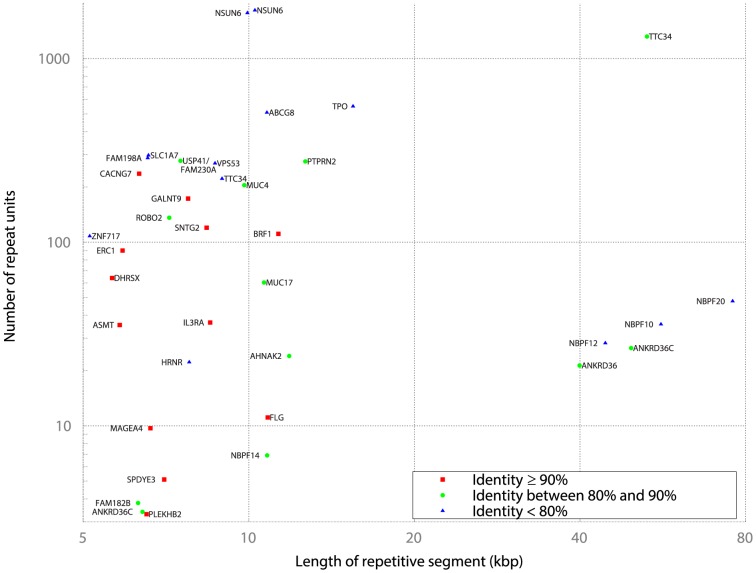
Genes with long internal repeats. The x-axis denotes the total length of the tandem repeat (log-scale), and the y-axis represents the number of repeat units within the tandem repeat (log-scale). The degree of repeat identity reported by TMRF is indicated by the color of the data point. Genes in bold have exonic sequence overlapping the repeat. Genes containing multiple disjoint long repeats appear more than once.

Using the Tandem Repeat Finder [63] (TMRF) track of the UCSC Genome Browser [64], I queried the database for protein-coding genes whose DNA sequence spans STR sequence, and filtered the results as described in the Methods. Figure 1 shows all 37 gene-internal repeats longer than 5 kb. *NSUN6*, *TTC34*, and *ANKRD36C* each contain multiple long repeats, and thus appear more than once. As previously discussed, high repeat length, high repeat count, and high repeat identity are markers of repeat mutability. Additionally, for intronic repeats, longer repeats are more likely to induce long mutations that in turn are more likely to alter methylation and splicing. At this scale, all repeats are minisatellites with intermediate to long repeat units.

Among the eleven genes with longest repeat length are thyroid peroxidase (*TPO*); protein-tyrosine phosphatase, receptor-type, n, polypeptide 2 (*PTPRN2*); and filaggrin (*FLG*). *TPO* encodes a primary autoantigen in both Hashimoto's Thyroiditis (HT) and Graves' Disease (GD); *PTPRN2*, and *FLG* encode primary autoantigens in Type-1 Diabetes (T1D) and Rheumatoid Arthritis (RA) respectively (Table 1). The presence of three primary autoantigens among the top eleven genes is highly significant (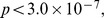 see Methods).

Additionally, the tenth ranked gene, *BRF1*, encodes an RNA-Polymerase-III (RNAP-III) initiation factor that binds to RNAP-III [65]. RNAP-III is an autoantigen specific to Systemic Sclerosis (SSc) (Table 1); *BRF1* thus encodes a peri-antigen for SSc.

### Autoimmune Associations of Genes with Long High-Identity Tandem Duplications

Motivated by known somatic mutation of *HP*, I looked for examples of long tandem duplications with at least 96% identity occuring within protein coding genes. Since the tandem repeat finder algorithm limits repeat units to 2000 bp, and its coverage of some longer units appears to be incomplete (e.g., it misses the 1.7 kb repeat in *HP*), such repeats may have been overlooked in the earlier analysis. The segmental duplications track of the UCSC database [64] was used as described in the Methods.

Table 3 shows all tandem duplications of total length at least 3400 bp where at least one duplicon occurs entirely within a protein-coding gene locus, and the tandem duplicons have the same orientation and are separated by at most 100 bp. Several genes appearing in Figure 1 also appear in Table 3, having long segments that are high identity tandem repeats. Of the remaining genes, five are autoantigens: complement component receptor 1 (*CR1*) in SLE and multiple sclerosis (MS); pepsinogen 4, group I (*PGA4*) in pernicious anemia (PA); titin (*TTN*) in myasthenia gravis (MG); interferon-gamma-inducible protein 16 (*IFI16*) in RA, SLE, SSc and SJ; and *HP* in celiac disease (CEL) (Table 1). The presence of five autoantigens among the top 33 genes is statistically significant (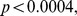 see Methods).

**Table 3:** Long tandem duplications with at least 96% identity that occur within a gene locus.

Gene	Length	Copies	Gap	Coding
*NBPF20*	76181	2 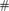	55	Y
*NBPF8*	65137	2	0	Y
*CR1*	54708	3	0	Y
*ANKRD30A*	47663	2	2	Y
*RBMY1A1*	47081	3	0	Y
*NBPF12*	44119	2 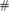	31	Y
*PGA4*	37662	2	0	Y
*TRPM3*	35986	2	0	N
*FCGBP*	31945	2 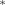	0	Y
*NEB*	31782	3	0	N
*NKG2-E* 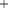	30864	2	0	Y
*TBC1D3C*/*TBC1D3H*	27063	2	0	N
*HCAR1*	26136	2	14	Y
*TTC34*	22675	2 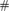	22	N
*DAZ1*	21690	2	0	Y
*NBPF1*	12620	2	17	Y
*NBPF12*	12568	2 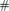	4	Y
*BRF1*	11321	2 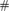	0	N
*C2orf78*	10103	2	58	Y
*CLEC17A*	8924	2	0	Y
*TTN*	8521	2	0	Y
*SNTG2*	8383	2 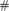	1	N
*IFI16*	8282	2	0	Y
*MUC5B*	7627	2 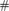	1	Y
*SPDYE3*	7020	5	0	Y
*ERC1*	5850	2 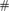	23	N
*HRNR*	5637	2 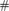	0	Y
*ACRC*	4289	2	66	Y
*SPRN*	4144	2	0	N
*TMEM132D*	3907	2 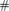	12	N
*HP*/*HPR*	3431	2	4	Y

Duplications were identified as described in the Methods. The length indicates the total length of the high-identity tandem duplicons. The gap is the separation between the two highest-identity (long) duplicons, which was required to be less than 100 bp. The duplication is “coding” if a duplicon overlaps at least one exon.

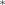
FCGBP has a third duplicon, but with less than 96% identity.

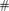
These genes have duplicons that are themselves STRs of lower fidelity; only the copy number for the high-identity long tandem duplication is reported in this table.

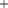
The segmental duplication containing NKG2-E overlaps the three genes KLRC1, KLRC2 and KLRC3.

Copy number variations in the 54.7 kb STR of *CR1* (Table 3) have been associated with SLE [66] and Alzheimer's disease (ALZ) [67]. The *CR1-S* allele has three repeats (as in the human reference genome) and has a population frequency of about 15%, while the shorter *CR1-F* allele has two repeats and a frequency of 83% [67]. The repeat length is functionally important, since the repeat includes sequence that codes for complement binding sites [67]. In both SLE and ALZ, the longer *CR1-S* allele is the high-risk variant [66], [67]. *CR1* plays an important immunological role in various cell types [68].

*PGA4* is one of three genes in the human reference genome coding for highly similar (but not identical) versions of pepsinogen A, an autoantigen in PA. Low levels of pepsinogen A are specific in diagnosing PA [69]. Variant alleles observed in the population contain three, two or one pepsinogen A gene [70]. The other major autoantigen in PA is *ATP4A*/*ATP4B* (Table 1), which both interacts with and colocalizes with pepsinogen A on the parietal cell surface [71].

The *HP* gene that has been observed in vivo to be somatically mutated [11] also codes for zonulin in individuals carrying the *HP2* allele [72]. The functions of haptoglobulin and zonulin are diverse, including some specific immunological capabilities conferred by the *HP2* allele [72], [73]. *HP2* alleles are overrepresented in several autoimmune diseases, coronary artery disease, and mental disorders [73]–[77].

### Additional Long Repeats Obtained from Self-Chain Alignments

To ensure completeness of the long repeat dataset, I queried the self-chain track of the UCSC database as described in the Methods. These alignments capture tandem repeats that may be slightly imperfect, i.e., there may be gaps between segments in the alignments, as well as repeats whose unit length is above the 2 kb threshold for TMRF. The results, shown in Table 4, are largely in agreement with Figure 1 and Table 3. Table 4 includes the following additional genes with alignments over 13 kb and exhibiting germ-line structural variation (File S1): *LPA*, *DMBT1*, *MGAM*, *KIR3DL1*, *KATNAL2*.

**Table 4:** Long (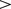
5 kb) regions of self-alignment within protein-coding genes.

Gene	Length	Gene	Length
*NBPF10*	45133	*MTUS2*	10090
*ANKRD30A*	40083	*ANKRD36*	8739
*NBPF20*	39623	*PTPRN2*	8649
*DAZ2*	38211	*FAM153A*	8495
*DAZ1*	36567	*TTC34*	8343
*LPA*	35017	*FAM153B*	7969
*NBPF12*	32343	*FLG*	7934
*FCGBP*	30167	*BRF1*	7650
*DMBT1*	26579	*ST3GAL4*	6583
*MGAM*	24595	*TTN*	6447
*DAZ4*	23181	*MUC12*	6346
*KIR3DL1*	22943	*MUC5B*	6303
*NEB*	20252	*GALNT9*	6290
*ANKRD30B*	18603	*TRHDE*	6161
*NBPF8*	14249	*ERC1*	5794
*TBC1D3C*	13432	*ROBO2*	5789
*TBC1D3H*	13432	*TM4SF2*	5498
*NBPF1*	13424	*NBPF14*	5345
*KATNAL2*	13368	*CACNG7*	5304
*CR1*	12971	*SNTG2*	5229
*HCAR1*	12648	*TNXB*	5227
*POTEJ*	12480	*MAGEA4*	5091
*DAZ3*	12115	*ASMT*	5021

Sequences with a self-similarity score of 60 or above having both query and target mapped within a protein-coding gene locus were obtained from the self-alignment track [267] of the UCSC database as described in the Methods, and ranked by match-length. In this table, the match length corresponds to the length of identity between the two duplicons. Note that self-aligned duplicons may overlap.

### *TTC34* is a Candidate CD4+ T Cell Antigen for Systemic Lupus Erythematosus

The gene *TTC34* is an outlier in Figure 1, both in terms of the length of the repetitive segment (an underestimate because the repeat is terminated by a gap in the human reference assembly) as well as the number of repeat units. *TTC34* encodes an uncharacterized protein that binds to PPP4C [78]. In support of a functional role for TTC34/PPP4C binding, RNAi depletion of either protein induces a common elongated cell phenotype [79].

If somatic mutation of *TTC34* induces autoimmunity, then antibodies to binding partners of TTC34/PPP4C would be expected. PPP4C is a ubiquitous serine/threonine phosphatase that regulates a variety of cellular functions [80]. Based on the localization of those cellular functions, I hypothesize that *TTC34* mutation underlies the initial pathenogenesis of SLE. Table 5 shows that many autoantigens in SLE, including known primary SLE autoantigens, associate with PPP4C. Under this hypothesis, the broad array of autoantigens in SLE is a consequence of the many functions of PPP4C, together with secondary immunogenicity caused by the aberrant clearance of apoptotic cells [52], [81].

**Table 5:** Correspondence of PPP4C localization with many known SLE autoantigens.

Autoantigen(s)	Putative PPP4C function/localization
**SNRPA**, SNRPC, SNRNP70, Sm	These are spliceosome proteins. PPP4C is involved in spliceosome assembly [80], [395].
SR proteins	SR proteins associate with the spliceosome [396]. See SNRPA, SNRPC, SNRNP70, Sm.
chromatin/histones	PPP4C binds to HDAC3, a histone deacetylase [397]. A PPP4C complex dephosphorylates 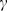 -H2AX histones [398].
ds-DNA	Stabilisation of stalled replication forks [80], histone deacetylation [397], histone dephosphorylation [398].
Ku70, Ku80	Ku70 and Ku80 associate with 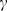 -H2AX histones during double strand break repair, mediated by DNA [399], [400]. A PPP4C complex dephosphorylates 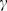 -H2AX histones [398].
PARP1	PARP1 binds with Ku [401]. See Ku.
**ribosomal P**	dephosphorylated during apoptosis by a caspase-induced phosphatase [402].
La(SSB)	La is dephosphorylated during apoptosis by a caspase-induced PP2A-like phosphatase [403].
**Ro60(TROVE2)**	See La; Ro60 and La are components of a common protein/RNA complex [404].
**APOH/cardio-lipin-complex**	APOH (coding for beta 2 glycoprotein I) associates with ANXA2/TLR4/CALR/NCL complexes [405]. Anti-APOH antibodies target bound APOH, triggering NF-Kappa B activation in a TRAF6/MyD88 dependent fashion in endothelial cells [405]-[407]. PPP4C physically interacts with TRAF6, and is recruited to the TLR4 complex on lipopolysaccharide (LPS) stimulation [408]. Further, LPS stimulation induces expression of PPP4C [408].
VIM/cardiolipin-complex, VIM	Vimentin is also observed in analysis of APOH/ANXA2/TLR4/CALR/NCL complexes [405]
NPM1	NPM1 binds cardiolipin [350]. See APOH/cardiolipin-complex.
CALR	CALR may be dephosphorylated by an okadaic-acid-sensitive protein phosphatase [409]. See La; CALR interacts with the Ro60/La/RNA complex [410]. See also APOH/cardiolipin-complex.
Ro52(TRIM21)	See CALR; Ro52 and CALR are binding partners [410].
NCL	See APOH/cardiolipin-complex.
ANXA2	See APOH/cardiolipin-complex.
F2/Phosphatidyl-serine, F2	Phosphatidylserine bound by ANXA2 [411]. See ANXA2.
ANXA1	Binds ANXA2 [412], phosphatidylserine [413], and colocalizes with ANXA5 [413]. See ANXA2, F2/Phosphatidylserine.
ANXA5	Binds phosphatidylserine as a monomer or dimer [414] and colocalizes with ANXA1 [413]. See ANXA1, F2/Phosphatidylserine.
HMGB1	Binds phosphatidylserine [415]. See F2/Phosphatidylserine.
LTF	Binds to TLR4 and activates the TRAF6/MyD88 pathway [416]. See APOH/cardiolipin-complex.

Primary autoantigens are in bold.

The long *TTC34* STR appears (with shorter length) in several primate species, but not in more distantly related species whose genomes have been sequenced [64]. Surprisingly, a 12 kb long STR has independently evolved in the mouse (GRCm38) genome, 3.2 kb upstream of the mouse *Ttc34* start site [82]. The mouse repeat unit length is 37, similar to the unit length of 40 in the human repeat. As for humans, the 12 kb mouse repeat is an outlier within the mouse genome: among all STRs that overlap a protein-coding gene locus, including a 5 kb segment upstream of the gene, the *Ttc34* repeat is the fifth longest (Table 6). The independent evolution of such a similar long repeat argues strongly for a functional role.

**Table 6:** Murine long (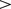
8 kb) STRs overlapping protein-coding RefSeq gene loci, including 5 kb upstream of the gene start site.

Gene	Largest Repeat length (bp)	Smallest unit size
*Ulk4*	19480	10
*St3gal4* 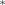	19106	1236
*Dmd*	18426	734
*Flg2*	16228	234
*Ttc34*	12289	37
*Hrnr*	8023	513

The smallest repeat unit for each region is given together with the total STR length. The *Ttc34* repeat ends 3.2 kb upstream of the gene start site.

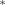
Two adjacent repeats reported by TMRF have similar repeat structure, and have been combined.

If the *TTC34* repeat mutates under inflammation [31], then the desired functional role would be one where changes in *TTC34* expression and/or PPP4C activity would be adaptive under inflammation. PPP4C depletion makes T cells resistant to apoptosis [83]. The association of apoptosis reduction with inflammation is biologically plausible, since T cells in inflammatory environments would be expected to receive survival signals during normal immune responses.

### *LPA* in Atherogenesis

*LPA* encodes a protein that binds to ApoB-100 in LDL particles to form Lp(a) lipoprotein particles containing lipids, phospholipids and cholesterol [84]. In coronary artery disease (CAD) ApoB-100 and LDL are immune targets of T cells and antibodies [85], meaning that *LPA* encodes a peri-antigen for CAD. Under the coherent somatic mutation hypothesis, rare but regular somatic mutation to *LPA* would occur, analogously to that observed for *HP* [11]. Epitopes of the mutant protein would be presented by immune cells in blood vessels, leading to activation of immune cells in atherosclerotic lesions [85] and autoimmune responses against other components of Lp(a) lipoprotein particles. *LPA* is central to CAD pathenogenesis, since an elevated plasma Lp(a) lipoprotein level predicts stroke and vascular disease, particularly in men [86], [87]. SNPs in *LPA* have the largest known effect on CAD risk [88], including an odds ratio of 1.74 for the minor allele of rs3798220.

### *ABCG8* in Hypercholesterolemia

**Figure 2:**
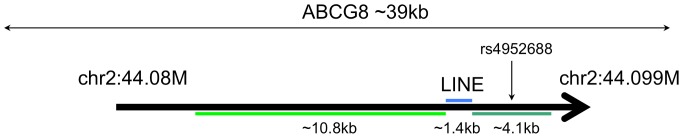
Structure of the long *ABCG8* repeat in the human reference genome. A 10.8 kb repeat and a 4.1 kb repeat have closely related repeat unit sequence, and are separated by a 1.4 kb LINE insertion. The SNP rs4952688 occurs in the middle of the 4.1 kb repeat.

*ABCG8* contains a long (10.8 kb) intronic repeat, part of a larger compound repeat separated by a LINE insertion (Figure 2). *ABCG8* encodes a cholesterol transporter that has been implicated in CAD [88], [89] and in gallstone formation [90]. SNPs rs41360247 and rs4245791 in *ABCG8* are associated with both CAD risk and LDL cholesterol levels [89]. Additionally, the SNP rs4952688 was shown to influence the mRNA expression of both *ABCG8* and its co-transporter *ABCG5* in liver cells [91]. rs4952688 is located within the compound repeat sequence (Figure 2), implicating this repeat sequence (or nearby linked sequence) in the expression levels of these two cholesterol transporters.

The normal function of *ABCG8* and *ABCG5* in liver cells is to excrete cholesterol into the bile [92]. Disruption of this process could lead to hypercholesterolemia, the initial manifestation of atherosclerosis. *ABCG8* variants can also influence cholesterol levels by modulating cholesterol absorption [93]. Somatic repeat mutations accumulating over time could change expression levels of these proteins, thereby altering the rate of cholesterol excretion/absorption. Germ-line mutations in these genes are associated with premature atherosclerosis [91], [94], as are mutations in other cholesterol transporters such as *APOE* [95], [96].

In principle, somatic repeat mutations could induce the production of aberrant ABCG8 protein variants that would be immunogenic, as previously argued for autoimmune disease. Antibodies to such variants could interfere with cholesterol excretion, but ABCG8-specific antibodies have not been documented in CAD. The molecular mechanisms by which the proteins encoded by *ABCG8* and *ABCG5* transport cholesterol are not fully understood [97]. If the ABCG5/ABCG8 complex binds to LDL, then *ABCG8* would encode a peri-antigen for CAD since oxidized LDL is an autoantigen [85].

### *DMBT1, FCGBP*, and the Mucins *MUC4, MUC5B, MUC12* and *MUC17*

Mucins including MUC4, MUC12 and MUC17 are important for intestinal integrity and have previously been associated with both ulcerative colitis (UC) and Crohn's disease (CD) [98]–[100]. MUC17 depletion increases epithelial permeability in the face of E. coli exposure [101]. FCGBP is a component of the mucus layer coating of the intestinal tract [102], and expression is higher in several autoimmune diseases [103]. The DMBT1 protein also provides mucosal protection of the intestine, and expression levels correlate with disease activity in CD and UC [104]. Host-microbe interactions appear to be central to the pathogenesis of UC and CD [105]. CD, UC, psoriasis (PSO) and ankylosing spondylitis (AS) have common features [105], [106] that suggest a cluster of diseases with related etiology. AS has been associated with the gut microbiome [107], and PSO has been associated with intestinal yeast infections [108].

A critical clue is provided by the *PTPN22* rs6679677 C/A polymorphism that is in high linkage disequilibrium with the rs2476601 C/T polymorphism associated with many autoimmune diseases [109]. At rs6679677, the A allele appears to be a risk allele for UC (as for most other autoimmune diseases) but protective for CD [105]. In the context of the coherent somatic mutation hypothesis, one could interpret this opposite *PTPN22* association in terms of alternative responses to somatic mutation. UC would be caused by an autoimmune response against the mutant protein, while CD would be caused by the failure of the mutant protein's function, in the absence of a direct immune response against that protein. This interpretation is consistent with a clear role for MHC alleles in UC but not CD [105], [110], and with a reduction in mucus quantity and/or goblet cell density specifically in UC [111], [112].

CD and UC have opposite risk alleles for *NOD2* polymorphisms [105]; *NOD2* variation modulates adaptive immune responses to microbial antigens [113], and regulates *DMBT1* expression in CD [114]. Significantly, short alleles of the *DMBT1* tandem repeat that encode fewer bacterial recognition sites are overrepresented in CD but not UC [104]. DMBT1 has high protein homology with the CD autoantigen CUZD1 [115], potentially leading to cross-reactive antibodies. Further, *DMBT1* -coded protein binds to pancreatic amylase [116], [117] that in turn binds to the CD autoantigen GP2 [118], meaning that *DMBT1* encodes a peri-antigen for CD.

In Sjogren's sydrome, a primary initiating change is the dysregulation of mucins [119], including the aberrant exocytosis of MUC5B [120]. *MUC4* is an interesting somatic mutation candidate because its expression pattern in the eye, vagina, ectocervix, trachea, and salivary gland [121] closely aligns with locations where symptoms occur [122]. *MUC5B* is expressed in many of these tissues [123], but not in the tear fluid [124]. Somatically mutated mucins could induce an immune response against the mutant protein. Alternatively, aberrant mucin protein may offer reduced protection of epithelial cells, making them vulnerable to infection. Apoptosis of the epithelial cell could trigger the induction of antibodies to apoptotically generated proteins in Sjogren's syndrome.

### Long Repeats Reside in Genes Expressed in Immune Cells and Implicated in Autoimmunity

*KIR3DL1* encodes an inhibitory receptor expressed on natural killer (NK) cells and T cells [125]. There is a high degree of copy number variation of the KIR genes around this locus, and some haplotypes do not possess *KIR3DL1* [126]. HLA-Bw4 is the ligand for KIR3DL1, and is protective in MS [125] and primary sclerosing cholangitis [127]. The presence of *KIR3DL1* is protective for AS [128], particularly AS with uveitis (UV) [129]. Somatic mutations to *KIR3DL1* could reduce inhibition of NK cells and/or T cells, leading to selective activation and clonal expansion.

The segmental duplication at the *NKG2-E* locus overlaps the genes *KLRC1*, *KLRC2* and *KLRC3*. Copy number variation at *NKG2-E* (manifested as a deletion of *KLRC2*) is associated with psoriasis susceptibility [130]. Reduced *KLRC2* expression in T cells is observed in PSO [131], and enhanced expression of *KLRC2* on CD4+ T cells is observed in MS [132]. *KLRC1* encodes a critical receptor on NK cells, regulating the elimination of autoreactive CD4+ T cells in animal models of MS [133]. *KLRC1* plays a critical role in tolerization by regulatory T cells [134], and is downregulated in PSO [135].

*KIR3DL1* and *KLRC1* encode NK cell receptors. NK cells and their receptors regulate autoimmunity in MS [136], and NK cell populations rise and fall in ways that correlate with the development of lesions in relapsing-remitting MS [137], [138]. NK cells are found in psoriatic plaques, and circulating NK cells are reduced in PSO, MS, SLE and T1D [139], [140].

The segmental duplication within the long *HCAR1* repeat identified in Tables 3 and 4 covers the two genes *HCAR2* and *HCAR3*. *HCAR2* codes for a niacin receptor that is expressed on antigen presenting cells and functions in a tolerization pathway for T cells [141]. Niacin administration ameliorates an animal model of MS through this pathway [141].

### Summary: Long Simple Tandem Repeats in Autoimmunity

Table 7 summarizes the autoimmune associations of genes with long STRs. This key table shows that long STRs within twenty genes are associated with sixteen common autoimmune diseases and atherosclerosis. Each of these putatively mutable STRs exhibits germ-line structural variation (File S1), consistent with a somatically mutable locus. The coherent somatic mutation hypothesis thus has the potential to be a comprehensive explanation for many autoimmune diseases.

**Table 7:** Known links between genes with long STRs and human autoimmune diseases.

Gene(s)	Disease	Antigen type	CNV	Expr. changes
*FLG*	RA	**Autoantigen**		
*TPO*	HT	**Autoantigen**		
*TPO*	GD	**Autoantigen**		
*PTPRN2*	T1D	**Autoantigen**		
*CR1*	SLE	Autoantigen	Yes	Yes
*CR1*	MS	Autoantigen		
*PGA4*	PA	Autoantigen		Yes
*TTN*	MG	Autoantigen		
*IFI16*	SLE, SSc, RA, SJ	Autoantigen		Yes
*HP*	CEL	Autoantigen	Yes	Yes
*BRF1*	SSc	Peri-antigen		
*TTC34*	SLE	Peri-antigen 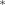		
*LPA*	CAD	Peri-antigen	Yes	Yes
*ABCG8*	CAD	Peri-antigen?		Yes
*DMBT1*	CD	Peri-antigen	Yes	Yes
*DMBT1*	UC			Yes
*MUC4*, *MUC12*, *MUC17*	CD, UC			Yes
*MUC5B*	SJ			Yes
*HP*	RA, SLE, CD, CAD, SSc		Yes	
*HP*	T1D		Yes	Yes
*FCGBP*	several			Yes
*KLRC2*	PSO		Yes	Yes
*KLRC2*	MS			Yes
*KIR3DL1*	AS, UV		Yes	

Genes with long STRs come from Figure 1, Table 3 and Table 4. A bold autoantigen label corresponds to a known primary autoantigen. The CNV column indicates whether a germ-line STR length variant is associated with the disease. Gene expression changes during disease are also shown.

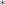
While many genes qualify as encoding peri-antigens in SLE, TTC34 encodes a peri-antigen for many autoantigens (Table 5).

With the exception of MS and possibly PA and SJ, each of the diseases associated with an autoantigen or peri-antigen in Table 7 is influenced by the functional rs2476601 single-nucleotide polymorphism in the *PTPN22* gene (Table 1). This polymorphism specifically influences T cell signaling [142], [143], B cell signaling [144], [145], autoreactive B cell generation [144], and T cell and dendritic cell hyper-responsiveness [146]. The role of *PTPN22* in some but not all autoimmune diseases suggests a common underlying pathway for this subset of diseases [45], [143] that may be related to STR length and/or mutability.

Table 8 shows that the conditions associated with autoantigens/peri-antigens above have a high degree of co-morbidity and/or familial association. Taken together, the data support the following model for this subset of diseases:

**Table 8:** Co-morbidity and/or familial associations between six autoimmune diseases and atherosclerosis.

	GD	RA	T1D	SLE	SSc	CAD
HT	[417]	[49], [418], [419]	[49], [418]	[49], [419], [420]	[419]	[421]
GD		[417]	[422]	[417]	[423]	[421] 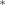
RA			[49], [418], [424]	[49], [424]	[49]	[421], [425]
T1D				[424]		[426]
SLE					[49]	[421], [425]
SSc						[421], [425]

Comorbidity may reflect common susceptibility factors or secondary disease effects, such as inflammation in RA contributing to CAD risk [427]. Comorbidities with some of these diseases exist for alopecia areata [428], [429], vitiligo [430], [430,431], juvenile idiopathic arthritis [432], myasthenia gravis [433], [434], and Addison's disease [435], five additional *PTPN22* -associated diseases, as well as celiac disease [436], [437] and pernicious anemia [438], [439].

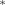
A link between GD and CAD is potentially confounded by the anti-atherogenic properties of thyroid hormones [440].

- For each gene containing a mutable repeat locus, individuals have a small population of somatically mutant cells.
- Under normal conditions, these mutant cells either induce peripheral tolerance or are too rare to trigger an immune response.
- Under inflammatory conditions (e.g., during an infection) the population of mutant cells increases, concurrently with immune system stimulation.
- In individuals with impaired tolerance or with sensitive B-cell or T-cell activation thresholds, reactions against mutant cells occur.
- Inflammation caused by immune reponses induces new coherent mutation in neighboring cells, and creates a cycle of autoimmunity.

A disjoint subset of diseases, including MS, PSO, UV, and AS have no association with the *PTPN22* gene polymorphism (Table 1). All four of these conditions are associated with immune-cell expressed genes spanning long repeats. Somatic mutation in those genes, rather than in antigenic genes, may be the critical step for such diseases.

### A Repeat Constituting 97% of the Intron Sequence within an Autoantigen for Pemphigus Vulgaris

Somatic repeat mutations in introns could be particularly disruptive when the intron is almost exclusively tandem repeat sequence. I therefore queried the reference genome for genes containing introns where a single tandem repeat occupies a large fraction of the intron (Table 9). The top-ranked gene in this analysis is *PKP3*, containing a 2310 bp repeat occupying over 97% of the eighth intron. There is germ-line structural variation at this locus in the HapMap population, with deletion variants encompassing almost the entire STR sequence [147].

**Table 9:** Genes with intronic tandem repeats occupying more than 90% of an intron.

Gene	Chrom.	Intron start	Intron end	Int. length	Rpt. Length	Occupancy	Copies
*PKP3*	chr11	400706	403076	2371	2310	0.974	155.9
*NMRK2*	chr19	3937287	3938599	1313	1274	0.970	27.8
*HSD17B14*	chr19	49337616	49339060	1445	1401	0.970	38.1
*PPP1R12C*	chr19	55604602	55605711	1110	1066	0.960	29.4
*ASMT*	chrX	1755454	1761694	6241	5827	0.934	35.4
*TCF25*	chr16	89973731	89975372	1642	1526	0.929	43.8
*NSMF*	chr9	140344708	140346815	2108	1948	0.924	33.4
*SCNN1D*	chr1	1223418	1225649	2232	2055	0.921	110.9
*AHNAK2*	chr14	105407316	105420216	12901	11844	0.918	24
*BRF1*	chr14	105695251	105707600	12350	11322	0.917	111
*TOP1MT*	chr8	144403557	144406167	2611	2367	0.907	164.1

*PKP3* encodes an autoantigen in pemphigus vulgaris (Table 2). Furthermore, PKP3 binds in vivo to several other primary pemphigus vulgaris autoantigens including DSG3, DSG1, DSC1, and DSC3 [148]. Aberrant PKP3 could therefore serve as a CD4+ T cell antigen in the induction of antibodies to these other proteins. The 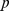 value for the top gene being an autoantigen is 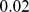 (see Methods).

### Genes with High Copy-Number Internal Repeats Include Autoantigens for Multiple Sclerosis and Myasthenia Gravis

**Figure 3:**
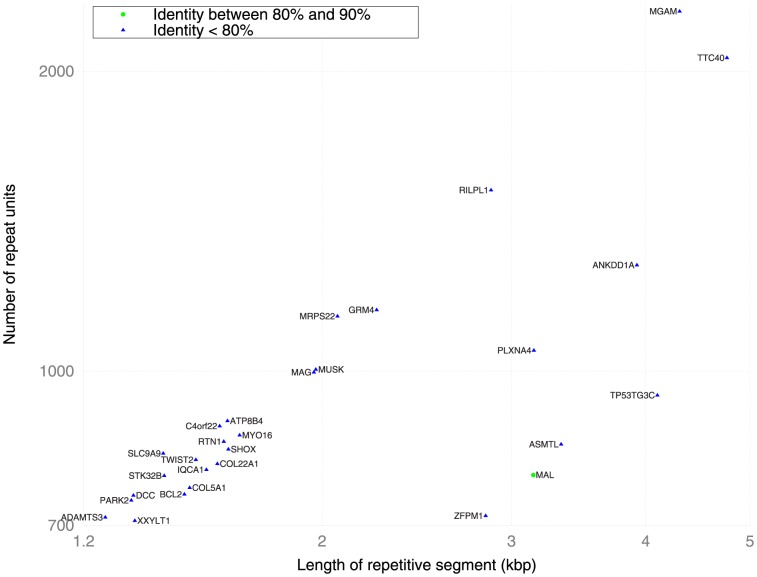
Genes with high copy number internal repeats. The x-axis denotes the total length of the tandem repeat (log-scale), and the y-axis represents the number of repeat units within the tandem repeat (log-scale). The degree of homology between repeat units is indicated by the color of the data point. All repeats in this diagram reside in introns. Genes containing multiple disjoint repeats appear more than once.

Figure 3 shows repeats of length up to 5 kb with repeat counts of at least 700 units. At this scale, all repeats are microsatellites with short repeat units. The genes with the eleventh and twelfth highest repeat counts genomewide are *MUSK* and *MAG* respectively. *MUSK* encodes an autoantigen in myasthenia gravis (Table 1). *MAG* encodes a multiple scleroisis autoantigen that binds in vivo to MBP and PLP [149], two other MS autoantigens (Table 1). Anti-MAG antibodies have also been observed in various polyneuropathies [150]–[152]. The presence of two autoantigens among the top twelve is statistically significant (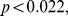 see Methods). On the other hand, the STRs in *MAG* and *MUSK* do not exhibit germ-line structural variation at 50 bp resolution (File S1); germ-line variation would be expected for a somatically mutable locus.

## Discussion

Somatic mutation has been overlooked or discounted as a cause of autoimmunity, primarily because “random” mutation would not lead to consistent and specific disease characteristics [153]. However, many kinds of somatic mutation are nonrandom, caused by mechanisms that yield coherent mutation patterns both within and across individuals. Coherent somatic mutation is a unifying and biologically plausible hypothesis to explain the specific targets of autoimmune disease.

### Longer-Range Segmental Duplications

**Figure 4:**
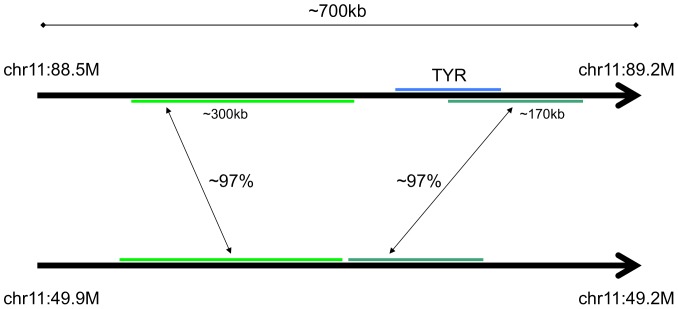
Structure of the *TYR* -related tandem duplications in the human reference genome. The long, high-identity duplicons make the region susceptible to gene conversion [274].

Long high-identity segmental duplications that are not strict tandem repeats may still lead to somatic protein changes via deletion or duplication if they partially overlap genes. Examples of this pattern include: RHD and GYPA, autoantigens in autoimmune hemolytic anemia; AMY2A, an autoantigen in autoimmune pancreatitis and fulminant T1D, and a binding partner of the CD autoantigen GP2 [118]; CES1 and PDIA3, autoantigens in type-2 autoimmune hepatitis; TYR, an autoantigen in vitiligo; and CHRNA7, an autoantigen observed in schizophrenia (Tables 1 and 2, [154]). The genomic structure of *TYR* makes it particularly susceptible to gene conversion and deletion (Figure 4).

The human genome contains segmental duplications that span whole genes, and copy number variation in these tandem repeats is likely to affect gene dosage [155]. These duplications are not considered in the primary anaylsis since repeat-dependent somatic mutation via deletion and/or duplication is less likely to induce altered protein. Nevertheless, the potential for altered protein exists through gene conversion or other processes that combine sequence from multiple instances of the gene. The primary autoantigen in Addison's disease is encoded by *CYP21A2* (Table 1), which resides within a segmentally duplicated region and is a known locus of germ-line gene conversion [156].

A five gene cluster (*GH1*, *GH2*, *CSH1*, *CSHL1*, *CSH2*) on chromosome 17 resides in a region characterized by complex segmental duplications with identity ranging from 92% to 96%. This cluster is a hot-spot for germ-line gene conversion [157]. Variations in these genes are associated with metabolic syndrome later in life [158]. Anti-pituitary antibodies are observed in conjunction with type-2 diabetes [159], [160] and GH1 is one of the autoantigens [161]. *GH1* codes for human growth hormone, and growth impairment is observed in celiac disease in conjunction with anti-pituitary antibodies [162].

### Mechanisms of Coherent Somatic Mutation

*PTPRN2* is an outlier not just in the length of its repetitive sequence; it has the most predicted sites of R loop formation in the whole genome [163]. The R loop sites do not overlap the 12 kb repeat in *PTPRN2*, but several long R loop sites occur about 20 kb upstream of this repeat. These R loops may contribute to the instability of the repeat region, and implicate mis-splicing [22] of *PTPRN2* in T1D.

Coherent somatic mutation can occur through a variety of mechanisms besides repeat instability and gene conversion, discussed below and summarized in Table 10.

**Table 10:** Multiple mechanisms generating coherent somatic mutation, and possible examples where autoimmunity results.

Mechanism	Possible Examples
Mutations at long tandem repeats	T1D, HT, RA, SLE, …
Gene conversion at segmental duplications	AD
Clonal expansion	Paraneoplastic autoimmune diseases, GD
Oxidative stress	*VIM* mutation in RA
RAG-dependent somatic mutation	*IKZF1* in RA
Pathogen Binding/Modification	*VIM* in RHF
Retrotransposition	BOMS
Apoptotic protein cleavage	Many cleaved proteins are autoantigens
Dysregulation of protein modification	Anti-TOP1 SSc
Environmental mutagens	*ENO1*, *VIM*, *FGB* in RA

### RAG-mediated Somatic Recombination and Rheumatoid Factor

Cancer studies provide valuable information about coherent somatic mutation in vivo. Many cancers elicit antibodies that are also found in autoimmune disease [164], further supporting a role for somatic mutation in autoimmunity. A striking example of coherent somatic mutation in cancer is the gene *IKZF1*. Internal *IKZF1* deletions occur in over 80% of cases of BCR-ABL1 acute lymphoblastic leukemia (ALL) [165]. Consistent breakpoints suggest aberrant RAG-mediated recombination [165]. The mutations coincide with a transition in the cancer from Chronic Lymphocytic Leukemia (CLL) to ALL.

CD5 expression on B cells is a common feature of both RA and CLL [166], CD5 expression correlates with RAG activity in B cells of people with autoimmune disease [167], and RAG is expressed in B cells in the RA synovium [168]. In RA, the appearance of rheumatoid factor (RF, an antibody to Fc-IgG) correlates with the hypogalactosylation of IgG, occuring roughly two years after the appearance of antibodies to citrullinated proteins, but two years before RA diagnosis [169]. RF is detected in several other autoimmune and infectious diseases [170].

If the RAG-dependent *IKZF1* mutations that consistently occur in ALL also occur in RA B cells, possibly followed by clonal expansion, then aberrant glycosylation would be explained because *IKZF1* appears to be critical for proper IgG glycosylation [171]. The improperly glycosylated IgG would be immunogenic. In the context of a normal immune response to a pathogen, a somatic mutation to *IZKF1* could be adaptive, because it would lead to RF production and potentially enhanced clearance of immune complexes [172]. However, in the context of an autoimmune response, RF production could increase the severity of disease [172]. RF is also found in SLE [173], and reduced *IKZF1* expression has been associated with SLE [174], [175].

### Mutagens and Oxidative Stress

Cigarette smoking is mutagenic, and appears to be selectively associated with antibodies to the primary autoantigens encoded by *ENO1* [176], *VIM* [177], and *FGB* [177] in RA. *VIM* mutations induced by oxidative stress influence antigenicity [178]. The association of RA with smoking is strong only among individuals with particular HLA alleles. A similar phenomenon occurs in MS [179]. This interaction of mutagen, autoantigen and HLA suggests that mutation is pathogenic primarily when the mutant epitope is well-presented by the corresponding antigen presenting molecules.

### Clonal Expansion Following Somatic Mutation

Somatic mutations in the *TSHR* gene are relatively common [180] and can induce activation and clonal expansion in thyroid tissue [181], [182], potentially explaining TSHR-antigenicity in GD. Paraneoplastic autoimmunity [164], [183], [184] is a related phenomenon in which an immune response to a tumor expressing mutant antigens also affects normal tissues expressing wild-type proteins.

### Pathogen-Induced Protein Binding and Modification

A pathogen-expressed protein that binds with an endogenous protein complex could serve as a CD4+ T cell target, providing help to B cells generating antibodies to proteins in the protein complex. A pathogen-modified endogenous protein could behave in a similar fashion

Rheumatic Fever (RHF) is a condition characterized by autoimmune attack against cardiac muscle, usually associated with group A streptococcal infections [185]. There is some in-vitro evidence of cross-reactivity of antibodies to streptococcal proteins and autoantigens in RHF [186]. Nevertheless, there is also evidence that mimicry may not be an important feature of RHF [187]. Autoreactivity to collagen in RHF has been proposed to result from collagen binding to streptococcal proteins [187].

The RHF autoantigens vimentin, myosin, and tropomyosin (Table 2) form part of the calcium-bound sarcomere protein complex [188]. Two lines of evidence implicate vimentin as an initiating autoimmune target (and peri-antigen) in RHF. First, vimentin is modified (ADP-ribosylated) by the group A streptococcal protein SpyA in a way that alters both its sequence and its organization [189]. Second, group A streptococci are known to bind to vimentin, particularly at sites of muscle injury [190].

### Apoptotic Cleavage

Adaptive immune reponses require the joint participation and mutual activation of CD4+ T cells and antigen-presenting cells such as B cells. B cells become anergic under chronic low-level exposure to antigen with limited costimulation [191]. Nevertheless, even anergic B cells can be activated with sufficient stimulation [191]. Protein that is post-translationally modified only upon apoptosis would presumably generate only low-level exposure to B cells. A post-translationally modified protein that forms part of a protein complex containing a somatic mutant is liable to trigger B cell/T cell co-activation. In such a case, a CD4+ T cell specific to the mutant peri-antigen could activate a previously anergic B cell clone. Such a mechanism could explain why post-translationally modified proteins, particularly those geneated during apoptosis, would be over-represented among B cell autoantigens [51], [52], [192].

### Retrotransposition

An additional potential mechanism of coherent somatic mutation is retrotransposition. Retrovirus [193], [194] and retrotransposon [195] integration hotspots exist, independent of selective pressure for cell growth/survival. This form of mutation could be relevant to Bout Onset Multiple Sclerosis (BOMS) in which an endogenous retrovirus has been implicated [196], [197], as well as schizophrenia [198] and amyotrophic lateral sclerosis [199]. Alternatively, retroviral expression could be a driver of neuroinflammation [200], leading to somatic mutation at other mutable repeat sequence.

### Dysregulation of Protein Modification Pathways

In SSc, the presence of one antibody type is generally exclusive of the others [201], [202], suggesting several subtypes of SSc with different mechanisms of induction. Chromosomal abnormalities are found at high frequency in the lymphocytes of patients with anti-centromere or anti-TOP1 antibodies, but at normal frequency in patients with anti-RNAPIII antibodies [203]. In SSc fibroblasts, increased sumoylation of TOP1 induces deficits in TOP1-mediated supercoiled-DNA relaxation [204] and disruption of TOP1 is known to cause chromosomal aberrations [203]. Inhibition of sumoylation improves TOP1 function in fibroblasts [203] and reduces fibrosis [205]. One interpretation of this data is that anti-TOP1 SSc is a sumoylation disorder. Hyper-sumoylated TOP1 could induce cell death via chromosomal aberrations, and at the same time trigger an immune response. Because the post-translationally modified protein would not be normally presented to immature B or T cells, tolerization to modified TOP1 would not occur. The centromere protein and SSc autoantigen CENPB is also a sumoylation target [206]–[209].

A similar neoantigen-creating role for sumoylation in a subset of patients with primary biliary cirrhosis (PBC) has previously been proposed [210]. In patients with antibodies to PML or SP100, two sumoylation target proteins [206]–[209], antibodies to SUMO2 and SUMO1 have been observed [210]. CENPB is also an autoantigen in PBC (Table 2). SSc and PBC are comorbid, with anti-CENPB as a common risk factor [211], [212], suggesting a shared etiology.

### Schizophrenia and Autism

Schizophrenia and autism have prominent immunological features, including HLA associations, comorbidity with autoimmune diseases, and associations with viral triggers and maternal infections during pregnancy (Table 11). Immunological theories of schizophrenia have been proposed [213].

**Table 11:** Immunological features of autism and schizophrenia.

Feature	Autism	Schizophrenia
HLA Association	Yes [441], [442]	Yes [442], [443]
Co-morbidity with autoimmune disease	Yes [441], [444]–[446]	Yes [447]–[449]
Viral triggers for disease	Yes [450]	Yes [450], [451]
Association with maternal infection during pregnancy.	Yes [441]	Yes [452]
Autoantibodies	Brain-specific antibodies in mothers and probands [441], [446], [453], [454]; Anti-nuclear antibodies [446], [455]	Yes [456]
Other	Gene expression changes reminiscent of autoimmunity [457]; NK cell dysregulation [458]; Amelioration of aberrant behaviors during fever [459]	Various immunological abnormalities [460]–[462]; differentially expressed genes involve immune pathways [463]

A clue that somatic repeat mutation may contribute to schizophrenia comes from a twin study in which a genomewide measure of somatic trinucleotide repeat mutation was obtained [214]. A high somatic trinucleotide mutation rate associated selectively with the schizophrenic proband in monozygotic twins discordant for disease [214].

Four *NBPF* family genes are among the top twelve in Figure 1, including the two longest STR sequences. The four *NBPF* genes in Figure 1 are located between positions 145.2 M and 148.3 M on chromosome 1, overlapping the 1q21.1 region. *NBPF* genes contain many copies of the *DUF1220* element; *DUF1220* copy number is closely related to brain size, and humans have many more copies than other primate species [215], [216]. In humans, high *DUF1220* copy number correlates with macrocephaly, and low copy number correlates with microcephaly [217], [218]. Germ-line deletions within the 1q21.1 region are associated with schizophrenia [219], [220], while duplications are associated with autism [217]. Somatic genomic instability is likely in such highly repetitive regions [217]. Somatic mutations early in embryonic development [221], suggested by the link to maternal infections during pregnancy, could lead to effects that mirror those of germ-line mutations. Early somatic mutation also creates the possibility that the thymus and brain express different haplotypes, preventing thymic deletion of T cells reactive to proteins coded by a brain-specific haplotype.

Other schizophrenia-associated genes among those in Figure 1 include *IL3RA* [222] and *CACNG7* [223]. *IL3RA* encodes a receptor for IL3 that is expressed in neurons, and IL3 expression is correlated with brain volume [222]. CACNG7 modulates neurite growth [224] and regulates AMPA receptor gating [225].

Several autism-related genes appear in Figure 1 and Table 4. SNTG2 binds to neuroligins 3 and 4, genes that have been associated with autism, and known autism-related mutations in those neuroligins weaken the binding with SNTG2 [226]. ROBO2 is an axon-guidance protein with significantly reduced expression in autistic brains [227]. *ASMT* encodes the last enzyme in the melatonin biosynthesis pathway, low melatonin expression is observed in autism spectrum disorders, and rare *ASMT* mutations are associated with autism [228]–[230]. *MGAM* is a gene involved in starch metabolism, with dysregulated mRNA expression in autism [231]. Germ line loss-of-function mutations in *KATNAL2* have been associated with autism [232].

Additional autism related genes appear in Figure 3 and exhibit structural variation in their STR sequence (File S1). Like ROBO2, PLXNA4 is an axon-guidance protein with significantly reduced expression in autistic brains [227]. ASMTL binds with TDO2 [233]; TDO2 is the rate-limiting enzyme in the catabolism of tryptophan, the precursor of serotonin, which is known to be elevated in 30% of autism cases [234].

There is a high concentration of autism-related genes among a relatively small set of putatively mutable genes. In light of the autoimmune features of autism (Table 11), this concentration suggests that somatic repeat mutation may contribute to the etiology of autism.

### Explaining Autoimmunity

A satisfying feature of the coherent somatic mutation hypothesis is that it provides a parsimonious yet comprehensive account of autoimmunity. The initiation of most diseases is attributed to a single mutable locus. A handful of diseases having several known subtypes include more than one corresponding mutable locus. Only four of the top sixteen genes in Figure 1 (*ANKRD36C*, *ANKRD36*, *AHNAK2*, *NSUN6*) do not have a link with an autoimmune disease, an autoimmune-associated mental disorder, or atherosclerosis. These relatively uncharacterized genes are promising candidates for future study.

The most prominent prior theory of autoimmunity is molecular mimicry, the hypothesis that peptides similar to host proteins are expressed by host-resident microbes, sometimes inducing an autoimmune reaction against the host proteins. The attractive feature of molecular mimicry has been that it provides a plausible explanation for the known link between infection and autoimmunity [235], [236]. However, despite decades of research, no human autoimmune diseases have been clearly attributed to molecular mimicry [235], [237], [238].

Autoimmune diseases have historically been categorized as organ-specific or systemic, with some diseases hard to categorize [239]. Under the coherent somatic mutation hypothesis, both kinds of disease have a common etiology, with the phenotype dependent on the expression patterns of the autoantigen. A narrow expression pattern (such as *PTPRN2*) leads to an organ-specific disease (T1D), while a widely expressed protein complex (TTC34/PPP4C as proposed in this report) leads to a systemic disease (SLE).

The incidence of each of several autoimmune diseases has been rising in recent years [240], as has the apparent incidence of autism [241]. The “hygiene hypothesis” states that autoimmune disease is linked to the absence of infections, through one of several possible immunoregulatory mechanisms [240]. Some infections that are protective if they occur early in development are possible triggers of autoimmunity if they occur later [240]. The present theory is consistent with a variant of the hygiene hypothesis in which tolerance to coherently mutated antigens is dependent on the early generation of such mutants. Infections or other inflammatory stimuli would increase the rate of somatic mutation, allowing for more efficient induction of peripheral tolerance. In the absence of peripheral tolerance, late generation of somatic mutants could induce autoimmunity. Alternative hypotheses based on increasing exposure to environmental mutagens [242], [243] are also consistent with an etiology dependent on somatic mutation.

### Autoinflammatory Disease

Several non-autoimmune diseases may also be caused by somatic mutation of highly mutable repeat sequence in the context of inflammation. Atopic dermatitis and icthyosis vulgaris are inflammatory skin conditions caused by inactivating germ-line mutations of the *FLG* gene in some cases [244], [245]. Somatic inactivating mutations of the 10.8 kb coding tandem repeat in *FLG*, reinforced by local inflammation, could contribute to the pathogenesis of these conditions. An accumulation of somatic mutations in *PTPRN2* (without autoimmunity) could lead to glucose intolerance [246]. Similar mechanisms could underlie various autoinflammatory conditions [247].

### Genetics

Our study is limited by its reliance on a single human genome for long repetitive sequence. Some reference alleles are much shorter than those typically observed in the population (e.g., *MUC1* [248], [249]). It is likely that long repetitive sequence is highly variable in the population [37], [38], [250], and that variations in germ-line sequence would modulate disease risk as seen for *CR1*, *LPA*, *HP* and *DMBT1*. Nevertheless, primary autoantigens whose genes contain long repeats were identified in a presumably healthy random individual, suggesting that, at least for those genes, all humans have some degree of somatic mutation and risk for disease.

Linkage based analysis of sequence variation in a population would not identify mutable repetitive regions because the high germ-line mutation rate would rapidly eliminate any linkage disequilibrium with adjacent sequence [157]. In contrast, there are likely to be few germ-line mutations within a pedigree, meaning that estimates of heritability [251] will include any effects of commonly inherited mutable sequence. Together, these effects could explain at least some of the missing heritability observed in many genomewide association studies [252]–[254].

### Immunological Aspects

Not all somatic mutation is likely to be immunogenic, even in protein-coding sequence. Somatic mosaicism observed in triplet repeat expansion diseases [255] would not generate immunogenic protein if the repeat length is longer than the fragment expressible in MHC molecules (8–10 amino acids for MHC-I, 15–24 amino acids for MHC-II). On the other hand, a long triplet repeat could be vulnerable to somatic deletions, yielding a short, potentially immunogenic peptide repeat.

Keratinocytes express *FLG* [256] and are non-professional antigen presenting cells (APCs) [257]. Pancreatic beta cells express *PTPRN2*, and thyroid epithelial cells express *TPO*; both of these cell types are also non-professional APCs. The purpose of antigen presentation by such cells is assumed to be tolerization in the absence of costimulatory molecules [258], which seems appropriate in the case of three primary (and putatively mutable) autoantigens. The presence of antigen presentation on these cell types may have allowed the evolution of mutable genes without significant risk of abrogating tolerance. Alternatively, antigen presentation within these cell types may have evolved as a response to selective pressure for longer repeat sequences in these genes.

While T cell tolerance can be induced by the administration of peptides [259], [260], attempts to induce tolerance in humans suffering from autoimmune disease have been largely unsuccessful [261]. Nevertheless, the success of these attempts is critically dependent on the peptide sequence used. The coherent somatic mutation hypothesis suggests that for intronic repeats, the initial immunogenic proteins may be mis-spliced or truncated forms of a native protein. Peptides covering the splice or truncation boundaries of putative mutant protein would be natural candidates for tolerance induction.

### Validation

Many of the high prevalence diseases in Table 1 have been specifically associated with mutable antigens or peri-antigens in the present report. Some more speculative hypotheses for the involvement of somatic mutation in other diseases are presented in File S1. The proposed associations should be considered tentative, and subject to experimental validation. For reasons described previously and below, experimental validation may be technically difficult.

Recent sequencing advances have the potential to accurately sequence long repetitive regions [250]. Accurately sequencing many cells in search of rare somatic mutants will require significantly more effort, although new technologies will help [6]. Obtaining putatively mutated cells from sites of autoimmune damage is challenging, since such cells would be subject to immunological destruction as soon as the mutation occurs.

## Conclusions

The coherent somatic mutation hypothesis states that recurrent or clonal somatic mutation underlies the initiation of autoimmune disease. Long STR sequence is likely to be somatically mutable in vivo, motivating the present study. A highly significant association between three primary autoantigens (covering four autoimmune diseases) and long STR sequence was established. Additional autoantigens and peri-antigens were identified among genes spanning long STR sequence, and among genes with other known markers of somatic mutation. The work presented here could lead to a partial resolution of the mystery of why particular proteins are targets of autoimmune destruction [50]. Experimental validation of the specific predictions made here is the next step.

## Materials and Methods

Genome coordinates use the GRCh37 (hg19) sequence. Gene names use HGNC approved nomenclature. Queries were submitted to the UCSC MySQL database server [64] and processed as described below. The SQL queries can be found in File S1. Gene transcripts were required to be protein-coding according to GENCODE version 17 [262] or (for Queries 2 and 6) RefSeq [263].

### Identifying Genes with Intragenic Repeats

Query 1 was submitted to obtain genes containing long or frequent repeats. The output from this query was edited as follows:

- Genes not on the reference chromosomes were removed. Only one such gene (*MGC39584*/*AC018692.2* on chr4_gl000193_random) had length over 5 kb and none had a repeat count over 100.
- For genes occurring on both the X and Y chromosomes, only the X chromosome instance was retained.
- TMRF often generated multiple repeat candidates for a region with the periods of the candidates being multiples of the shortest period. In such cases, only the shortest-period candidate with the highest repeat-unit count was kept, even if it spanned a slightly smaller region.
- When TMRF generated a consensus repeat unit that was itself repetitive (e.g., AGTTAGTTAGTT) the TMRF entry was replaced by one with a shorter repeat unit (e.g., AGTT) and a higher repeat-unit count, retaining the degree of identity from the longer sequence. Examples include *VPS53* (in which a 96 bp repeat is itself made of 3 instances of a 32 bp repeat), *MUC4* (in which a 96 bp repeat consists of two consecutive instances of a 48 bp repeat), and *MAL* (with an 8 bp AGTGAGTG repeat).
- In a small number of cases, TMRF generated multiple essentially contiguous repeats with the same period and consensus sequence. The only such case where the repeat was either more than 5 kb long or contained more than 600 repeat units was *PTPRN2* (chr7:158122660–158135328) where the contiguous repeat records were combined into a single longer 12.6 kb repeat.

To see whether the output was dependent on the source of the gene annotations, I reformulated the query as Query 2 using RefSeq [263]. The following differences were noted for repeats longer than 5 kb:

- There was some discrepant labeling of the *NBPF* genes. The *NBPF* repeat sequences were the same, with the exception of one *NBPF10* repeat (see below).
- The following genes/repeat-lengths were identified by GENCODE but not RefSeq: *ANKRD36C*/49539; *FAM230A*, *USP1*/7516; *PLEKHB2*/6521; *ANKRD36C*/6410; *FAM182B*/6292.
- The following genes/repeat-lengths were identified by RefSeq but not GENCODE: *NBPF10*/15997; *ANKRD36B*/25486; *MUC19*/8607.
- A large majority of repeats were common to the two annotations, with the differences mentioned above largely due to differences in the labeling of a gene transcript as protein coding.

The differences between the two annotations appear to be small. The *MUC19* transcript identified by RefSeq may have immunological significance given the association of *MUC19* with Crohn's disease and ulcerative colitis [105], [264], [265].

Genes that span gaps in the human assembly where the gaps are presumed to include repetitive sequence (e.g., *MUC5AC* [250]) are absent from the query result. Applying the tandem repeat finder algorithm [63] to the *MUC5AC* exon 31 sequence reported by Guo et al [250] revealed a longest tandem repeat of 1.6 kb.

### Identifying Genes Spanning Long Segmental Duplications

Query 3 was used to identify a preliminary set of segmental duplications occuring within protein-coding genes, using the segmental duplication track [266] of the UCSC MySQL database server [64]. At least one duplicon was required to occur entirely within the gene sequence. The structure of the identified segmental duplications was examined using the UCSC genome browser. Where more than two contiguous tandem duplications exist (*CR1*, *NEB*, *SPDYE3*), the records for the gene were combined into a single record for the longer compound tandem repeat. When multiple segmental duplications overlapped (*TTC34*) only the longer duplication was retained.

### Additional Queries

Query 4 was used to identify long self-alignments (score at least 60) within protein-coding genes, using the self-alignment track [267] of the UCSC MySQL database server [64]. Query 5 was used to identify repeats constituting almost an entire intron within a gene. Query 6 was used to identify long repeats in the mouse genome; repeats are required to overlap a protein-coding RefSeq gene, including 5 kb of sequence upstream of the gene start site.

Query 7 was used to identify pairs of long repeats where the second repeat unit is the reverse complement of the first. The purpose of this analysis is to understand the genomewide significance of this feature of the *NSUN6* repeats (File S1). The output of this query was filtered to remove sequences on unplaced chromosomes and rows in which the two repeat sequences are not reverse complements. Queries 8 through 12 identify structural variation at STR loci utilizing information from the DGV database [268]–[270] (File S1).

### Significance of Autoantigen Over-Representation in Gene Lists

#### Primary Autoantigens

To determine the statistical significance of a set of primary autoantigens within a gene list, an estimate of the number of known primary autoantigens for common autoimmune diseases is required. Based on Table 1, there are nineteen known primary autoantigens for those diseases. This number includes pANCA, a category covering five proteins in UC [271], and ribosomal P (3 proteins), so a more precise estimate of the number of genes is 25. The null hypothesis 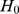 states that each gene associated with a primary autoantigen is equally likely to appear anywhere in the ranked list of genes. There are 20,330 protein-coding genes in GENCODE V17 [272]. Choosing the top eleven genes is therefore well approximated by a binomial process, where a selected gene has a 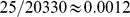 probability of being a primary autoantigen under the null hypothesis.

I apply an exact one-sided binomial test of goodness of fit. The *p*-value for 3 or more of the top 11 genes being primary autoantigens under the null hypothesis is 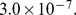 The significance is robust to the size of the prefix of the gene list. For example, taking the top 35 genes rather than the top 11 yields 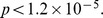 One can therefore reject the null hypothesis and conclude that the overrepresentation of primary autoantigens near the top of the list is highly significant.

#### Autoantigens

Determining the significance of a set of autoantigens within a gene list requires an estimate of the total number of autoantigens. Stadler et al. [54] tabulate 348 known autoantigens, but this list is incomplete (e.g., it does not include *FLG* or *PKP3*). For the purposes of determining a 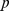 value, 400 autoantigens and 20,330 protein-coding genes [272] are assumed for a one-sided binomial goodness of fit test. All 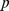 values calculated above remain significant at 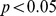 even if an estimate of 600 autoantigens was used.

## Supporting Information

File S1 (PDF)
