# Supplementary material for: Coherent Somatic Mutation in Autoimmune Disease
Source: PLoS One. 2014 Jul 2;9(7):e101093. doi: 10.1371/journal.pone.0101093 (PMC4079513; doi:10.1371/journal.pone.0101093)
Supplement: File S1 — (PDF) [file pone.0101093.s001.pdf]

K. A. Ross: Coherent Somatic Mutation in Autoimmune Disease.
Supporting Information.

Speculative Observations of Gene Function

Immunological Significance of *NSUN6*

NSUN6 has a distinctive repeat pattern in two respects. First, two long repeats of roughly equal length are adjacent. Second, the two repeat units are reverse complements. The resulting palindromes have the potential to form cruciform structures [S1], which lead to genetic instability [S2]. While the identity between palindromic segments is relatively low, the sequence remains palindromic when shifted by a multiple of five bases, giving many more opportunities for the formation of cruciform structures.

I queried the UCSC browser database to determine whether there were additional regions in the human genome with similar characteristics, i.e., (a) at least 2kb and at least 50 units in each repeat, (b) inverted orientation with at most 1kb between repeats, and (c) a repeat unit longer than 2bp but shorter than 100bp. Besides *NSUN6*, there are three such regions in the human genome (Table S-1), two of which are located in centromeric regions away from genes. The third example has the same pair of inverted 5bp repeat units as *NSUN6*, and is located on chromosome 2 at a position 11kb upstream of immunoglobulin kappa variable sequence [S3].

Table S-1. Human reference genome sequence containing pairs of STRs having: (a) at least 2kb and at least 50 units in each repeat, (b) inverted orientation with at most 1kb between repeats, and (c) a repeat unit longer than 2bp but shorter than 100bp.

Chrom.	Location	Unit size	Copies	Identity (%)	Sequence
10	39076595	5	7271.6	65	TTCCA
10	39113854	5	8212.8	66	TGGAA
2	89850110	5	4789.2	68	TTCCA
2	89874086	5	1178.4	73	TGGAA
10	18842234	5	1830.4	67	TGGAA
10	18852502	5	1770.8	67	TTCCA
22	16565087	48	82.9	75	GGGACAAAC...
22	16569067	48	68.6	73	GTTTGTCCC...

NSUN6 encodes a putative methyltransferase with unknown function. Curiously, the repeat in *NSUN6* is about 11kb away from the adjacent gene *CACNB2*. CACNB2 encodes an autoantigen in Lambert-Eaton myasthenic syndrome [S4] and a susceptibility locus for several mental disorders [S5].

GALNT9

The *GALNT9* gene contains a 7.7kb repeat with 94% identity (Figure 1). *GALNT9* encodes a protein responsible for O-glycosylation, with expression primarily in specific areas of the brain [S6], although expression in B cells [S7] has been reported. Toba et al [S6]. speculate that GALNT9 may be involved in the O-glycosylation of tenascin-R (TNR) and beta-amyloid precursor protein (APP). If so, then somatic mutation at *GALNT9* could influence processes that depend on the O-glycosylation of these proteins.

O-glycosylated TNR is a ligand for MAG [S8, S9], and is involved in myelination [S10]. Disruption of O-glycosylated TNR could be relevant to multiple sclerosis and other demyelinating diseases.

The maturation of APP involves the addition of several short O-glycans [S11]. O-glycosylation is an important step during APP cleavage [S12], and influences amyloid beta processing [S13]. Dysregulated O-glycosylation could be relevant to Alzheimer's disease, in which amyloid plaques are the central disease feature.

CLEC17A

The *CLEC17A* gene interacts with *BLNK*, a gene essential for B-cell receptor (BCR) signaling [S14]. CLEC17A appears to be responsible for recruitment of BLNK to the cell membrane [S14]. Somatic mutations to *CLEC17A* could alter the BCR signaling pathway in some B cells. If those B cells are autoreactive and selectively undergo expansion, autoimmunity could result.

Myelination Genes

Besides *MAG*, several other myelination-related genes have long tandem repeats or high repeat counts. *TRPM3* is a calcium-channel protein in oligodendrocytes that participates in central nervous system myelination [S15]; calcium channel perturbation is associated with MS [S16]. *GRM4* dampens the immune response in mouse models of multiple sclerosis [S17]. *MAL* is involved in myelination in both the central and peripheral nervous systems [S18].

Ankylosing Spondylitis

While there is no strong evidence of specific autoantibodies in AS [S19] the *ACAN* gene is an interesting candidate because it is a T cell immune target in AS [S20]. *ACAN* has a 1.8kb coding minisatellite that influences lumbar degenerative disc disease [S21].

Parkinson's Disease

Parkinson's disease (PD) is linked with inflammation and autoimmunity [S22–S24]. Two genes with potential relevance to PD are *RILPL1* (1519 repeat units) and *PARK2* (741 repeat units). RILPL1 is neuroprotective via binding to GAPDH [S25]; deprenyl, a PD drug, also binds to GAPDH to prevent death cascade induction [S26]. Mutations and copy number variants in *PARK2* [S27, S28], aberrant *PARK2* splicing [S29], and reduced *PARK2* expression [S30] are associated with PD. Another candidate gene is *SNCAIP*, a gene associated with Parkinson's disease, that is regularly somatically duplicated in medulloblastoma [S31].

Amyotrophic Lateral Sclerosis

Amyotrophic Lateral Sclerosis (ALS) also has links to inflammation and autoimmunity [S32–S34]. A gene with potential relevance to ALS is *VPS53*, which contains a long (8683bp) tandem repeat. *VPS53* encodes a subunit of the GARP complex [S35], and mutations in another GARP complex gene (*VPS54*) cause the wobbler mouse phenotype, reminiscent of ALS, with reduced *VPS53* expression [S36].

Germ-Line Variation at STR Loci

Repetitive sequence is a marker of germ-line variability [S37, S38]; actual variability can be verified by consulting data on structural variation. Wong et al. [S39] utilize high coverage data of 96 individuals to obtain genomewide data about structural variation in the Malay population. The high coverage (30X) data allowed roughly twice as many deletion variants to be detected as a low coverage (5X) version of the

data [S39]. When an STR locus exhibits structural variation within the STR boundaries, "internal" germ-line variation is apparent. Structural variation that spans an STR locus is termed "external" variation. Any reported difference from the reference sequence is considered variation. Only differences exceeding 50bp were reported by Wong et al. [S39].

Table S-2. Germ-line variability according to Wong et al. [S39] at each of the STR loci of Figure 1. The *NBPF* family of genes and several additional genes show only external variation; these STRs occur in regions of complex segmental duplications that induce larger-scale structural variation. Only *MAGEA4* and *ERC1* show no variation at all.

Gene	Length	Internal Variation	External Variation
NBPF20	75756		Y
NBPF10	56130		Y*
TTC34	52937	Y	
ANKRD36C	49539	Y	Y*
NBPF12	44470		Y*
ANKRD36	39925	Y	Y*
TPO	15472	Y	
PTPRN2	12668	Y	
AHNAK2	11843	Y	
BRF1	11321	Y	
FLG	10828	Y	
NBPF14	10798		Y
ABCG8	10788	Y	
MUC17	10659	Y	Y
NSUN6	10260	Y	
NSUN6	9943		
MUC4	9813	Y	
TTC34	8941	Y	
VPS53	8683	Y	
IL3RA	8509	Y	Y
SNTG2	8384	Y	
HRNR	7794	Y	
GALNT9	7757	Y	
USP41,FAM230A	7516	Y	
ROBO2	7169	Y	
SPDYE3	7020		Y
MAGEA4	6627		
SLC1A7	6572	Y	
FAM198A	6551	Y	
PLEKHB2	6521		Y
ANKRD36C	6410		Y*
CACNG7	6321		Y*
FAM182B	6292	Y	Y
ERC1	5898		
ASMT	5826	Y	
DHRSX	5644	Y	
ZNF717	5139	Y	

*These variants were uniform within the population studied by Wong et al. [S39] but different from the reference sequence. All remaining loci exhibited multiple alleles in the population.

Table S-3. Germ-line variability according to Wong et al. [S39] at each of the STR loci of Figure 3. A minority of the genes, mostly those with the highest repeat counts, show germ-line STR variability.

Gene	Repeat Count	Internal Variation	External Variation
MGAM	2296.5	Y	
TTC40	2062.5	Y	
RILPL1	1519	Y	
ANKDD1A	1277.3		
GRM4	1151.5	Y	
MRPS22	1135		
PLXNA4	1048.7	Y	
MUSK	1003.5		
MAG	997		
TP53TG3C	945.5		Y*
ATP8B4	891		
C4orf22	880.5		
MYO16	862		
RTN1	849.5		
ASMTL	844.2	Y	Y
SHOX	834.5	Y	
SLC9A9	826.5		
TWIST2	814.5		
COL22A1	807	Y	
IQCA1	796		
MAL	786.4		
STK32B	785		
COL5A1	763.5		Y*
BCL2	752		
DCC	750		
PARK2	741.5		
ZFPM1	715.5		
ADAMTS3	713		
XXYLT1	707.5		

*These variants were uniform within the population studied by Wong et al. [S39] but different from the reference sequence. All remaining loci exhibited multiple alleles in the population.

Table S-4. Germ-line variability according to Wong et al. [S39] at each of the STR loci of Table 3. While structural variation at *PGA4* is not observed in the population studied by Wong et al. [S39], extensive structural variation is observed at *PGA4* in other populations [S40]. Similarly, structural variation around the STR in *TTN* has been observed in other populations (e.g., [S41]).

Gene	Length	Internal Variation	External Variation
NBPF20	76181		Y
NBPF8	65137		
CR1	54708		Y
ANKRD30A	47663		Y
RBMY1A1	47081		
NBPF12	44119		Y
PGA4	37662		
TRPM3	35986		
FCGBP	31945	Y	
NEB	31782		
NKG2-E	30864	Y	
TBC1D3C/TBC1D3H	27063		
HCAR1	26136	Y	
TTC34	22675	Y	Y
DAZ1	21690		
NBPF1	12620		Y
NBPF12	12568		Y*
BRF1	11321	Y	
C2orf78	10103		
CLEC17A	8924	Y	
TTN	8521		
SNTG2	8383	Y	
IFI16	8282	Y	
MUC5B	7627		Y*
SPDYE3	7020		Y
ERC1	5850		
HRNR	5637	Y	
ACRC	4289	Y	
SPRN	4144		
TMEM132D	3907		
HP/HPR	3431		Y

*These variants were uniform within the population studied by Wong et al. [S39] but different from the reference sequence. All remaining loci exhibited multiple alleles in the population.

Table S-5. Internally variable STRs within long (>5kb) regions of self-alignment within protein-coding genes (Table 4) according to Wong et al. [S39]. Unlike Table S-2, internal variation is found in *NBPF10*, *ERC1* and *MAGEA4*. The self-chain boundaries are more permissive, allowing for gaps in the alignment. While variation within the STR in the *LPA* gene is absent for the population of Wong et al. [S39], structural variation within the *LPA* STR has been observed in other populations (e.g., [S41, S42]).

Gene	Length
NBPF10	45133
FCGBP	30167
DMBT1	26579
MGAM	24595
KIR3DL1	22943
ANKRD30B	18603
KATNAL2	13368
HCAR1	12648
POTEJ	12480
MTUS2	10090
ANKRD36	8739
PTPRN2	8649
TTC34	8343
FLG	7934
BRF1	7650
ST3GAL4	6583
MUC12	6346
GALNT9	6290
TRHDE	6161
ERC1	5794
ROBO2	5789
TM4SF2	5498
CACNG7	5304
SNTG2	5229
MAGEA4	5091
ASMT	5021

SQL Queries

Query 1 GENCODE V17 protein-coding genes containing long ($\geq$1000bp) repeats as applied to the hg19 dataset in the UCSC MySQL database. To rank by repeat length, the phrase `order by length desc` can be appended; to rank by repeat frequency, the phrase `order by copyNum desc` can be appended.

```
select distinct g.name2, s.*
from (select *, chromEnd-chromStart as length
     from simpleRepeat
     where chromEnd-chromStart>=1000) s,
     wgEncodeGencodeBasicV17 g,
     wgEncodeGencodeAttrsV17 a
where g.chrom=s.chrom and g.txStart<s.chromEnd
      and g.txEnd> s.chromStart and
      a.transcriptId=g.name and a.transcriptType='protein_coding'
```

Query 2 RefSeq protein-coding genes containing long ($\geq$1000bp) repeats as applied to the hg19 dataset in the UCSC MySQL database. The "NM" prefix indicates a protein-coding gene [S43].

```
select distinct g.name2, s.*
from (select *, chromEnd-chromStart as length
     from simpleRepeat
     where chromEnd-chromStart>=1000) s,
     refGene g
where g.chrom=s.chrom and g.txStart<s.chromEnd
      and g.txEnd> s.chromStart and
      g.name like 'NM%';
```

Query 3 Long tandem segmental duplications within GENCODE V17 protein-coding genes.

```
select distinct g.name2, otherStart-chromEnd as gap,
                chromEnd-chromStart+otherEnd-otherStart as totlen,
from (select *
     from genomicSuperDups
     where chrom=otherChrom and
     otherStart+1 between chromStart and chromEnd+101
     and fracMatch>=0.96 and strand="+") s,
     wgEncodeGencodeBasicV17 g,
     wgEncodeGencodeAttrsV17 a
where s.chrom = g.chrom and
      (((g.txStart<s.chromStart and g.txEnd>s.chromStart) and
      (g.txStart<s.chromEnd and g.txEnd>s.chromEnd)) or
      ((g.txStart<s.otherStart and g.txEnd>s.otherStart) and
      (g.txStart<s.otherEnd and g.txEnd>s.otherEnd))) and
      chromEnd-chromStart+otherEnd-otherStart > 3400 and
      a.transcriptId=g.name and a.transcriptType='protein_coding'
order by chromEnd-chromStart+otherEnd-otherStart desc;
```

Query 4 Query used to identify long self-alignments within GENCODE V17 protein-coding genes.

```
select g.name2, g.chrom, max(f.matchLen)
from (select *, score/normscore as matchLen
     from chainSelf
     where tEnd-tStart>=5000 and qstrand="+"
     and normscore >=60) f,
     wgEncodeGencodeBasicV17 g,
     wgEncodeGencodeAttrsV17 a
where g.chrom=f.tName and g.chrom=f.qName and g.txStart<f.tStart
      and g.txEnd>f.tEnd and g.txStart<f.qStart and g.txEnd>f.qEnd
      and a.transcriptId=g.name and a.transcriptType='protein_coding'
group by g.name2, g.chrom
having max(f.matchLen) > 5000
order by max(matchLen) desc;
```

Query 5 Query used to identify long tandem repeats (at least 1000bp) within introns of GENCODE V17 protein-coding genes. The intermediate query `n` contains all integers between 1 and 363 and is abbreviated below; the number 363 is the number of exons in the GENCODE transcript with the most exons.

```
select t.*, t.intronEnd-t.intronStart+1 as IntronLen,
       s.chromEnd-s.chromStart+1 as ReptLen,
       (1.0*s.chromEnd-s.chromStart+1)/(t.intronEnd-t.intronStart+1)
       as Occupancy
from (select distinct g.name2, g.chrom,
     CAST(REPLACE(SUBSTRING(SUBSTRING_INDEX(exonEnds,',',n.i),
          LENGTH(SUBSTRING_INDEX(exonEnds,',',n.i-1)) + 1),',', '')
          as UNSIGNED INTEGER) +1 as intronStart,
     CAST(REPLACE(SUBSTRING(SUBSTRING_INDEX(exonStarts,',',n.i+1),
          LENGTH(SUBSTRING_INDEX(exonStarts,',',n.i)) + 1),',', '')
          as UNSIGNED INTEGER) -1 as intronEnd
     from wgEncodeGencodeBasicV17 g, wgEncodeGencodeAttrsV17 a,
          ( select 1 as i union all
          select 2 union all
          ...
          select 363 ) n
     where a.transcriptId=g.name
           and a.transcriptType='protein_coding'
           and n.i < g.exonCount ) t,
     (select *
     from simpleRepeat
     where chromEnd-chromStart>1000 ) s
where t.chrom=s.chrom and t.intronStart <= s.chromStart
      and t.intronEnd >= s.chromEnd
order by (1.0*s.chromEnd-s.chromStart+1)/(t.intronEnd-t.intronStart+1)
         desc;
```

Query 6 Query used to identify long tandem repeats in the mouse (mm10).

```
select distinct g.name2, length, s.period
from (select *, chromEnd-chromStart+1 as length
     from simpleRepeat
     where chromEnd-chromStart>=1000) s,
     refGene g
where g.name like 'NM%' and g.chrom=s.chrom and (
      (g.strand='+' and g.txStart-5000<s.chromEnd
                    and g.txEnd> s.chromStart)
      or
      (g.strand='-' and g.txStart<s.chromEnd
                    and g.txEnd+5000> s.chromStart))
order by length desc;
```

Query 7 Query used to identify palindromic pairs of long tandem repeats.

```
select s.chrom, s.chromStart, s.chromEnd,
       s.period, s.copyNum, s.perMatch,
       s.length, s.sequence, t.chromStart,
       t.chromEnd, t.period, t.copyNum,
       t.perMatch, t.length, t.sequence
from (select *, chromEnd-chromStart as length
     from simpleRepeat
     where copynum>50 and chromEnd-chromStart>2000
           and period<100) s,
     (select *, chromEnd-chromStart as length
     from simpleRepeat
     where copynum>50 and chromEnd-chromStart>2000
     and period<100) t
where t.chrom=s.chrom and t.period=s.period
      and t.chromStart between s.chromEnd and s.chromEnd+1000
order by s.period asc, s.length desc;
```

Query 8 Query used to identify internal structural variation at STR loci using the data from Wong et al. [S39], whose PubMed identifier is "23290073".

```
select p.name2, p.chromStart, p.chromEnd,
       p.length, p.copyNum, p.perMatch, p.period,
       sum(sampleSize) as samples,
       sum(observedGains) as gains,
       sum(observedLosses) as losses,
       count(*) as cnt
from (select distinct g.name2, s.*
     from (select *, chromEnd-chromStart as length
          from simpleRepeat
          where chromEnd-chromStart>=1000) s,
     wgEncodeGencodeBasicV17 g,
     wgEncodeGencodeAttrsV17 a
     where g.chrom=s.chrom and g.txStart<s.chromEnd
     and g.txEnd> s.chromStart and a.transcriptId=g.name
     and a.transcriptType='protein_coding') p,
     dgvMerged d
where d.pubMedId="23290073" and d.chrom=p.chrom
      and d.chromStart>p.chromStart and d.chromEnd<p.chromEnd
group by p.name2, p.chromStart, p.chromEnd, p.length, p.copyNum, p.perMatch, p.period
```

Query 9 Query used to identify external structural variation at STR loci using the data from Wong et al. [S39].

```
select p.name2, p.chromStart, p.chromEnd,
       p.length, p.copyNum, p.perMatch, p.period,
       sum(sampleSize) as samples,
       sum(observedGains) as gains,
       sum(observedLosses) as losses,
       count(*) as cnt
from (select distinct g.name2, s.*
     from (select *, chromEnd-chromStart as length
          from simpleRepeat
          where chromEnd-chromStart>=1000) s,
     wgEncodeGencodeBasicV17 g,
     wgEncodeGencodeAttrsV17 a
     where g.chrom=s.chrom and g.txStart<s.chromEnd
     and g.txEnd> s.chromStart and a.transcriptId=g.name
     and a.transcriptType='protein_coding') p,
     dgvMerged d
where d.pubMedId="23290073" and d.chrom=p.chrom
      and d.chromStart<p.chromStart and d.chromEnd>p.chromEnd
group by p.name2, p.chromStart, p.chromEnd, p.length, p.copyNum, p.perMatch, p.period
```

Query 10 Query used to identify internal structural variation at long tandem repeat loci (Table 3) using the data from Wong et al. [S39].

```
select p.name2, p.chromStart, p.chromEnd, length, sum(sampleSize) as samples,
       sum(observedGains) as gains, sum(observedLosses) as losses, count(*) as cnt
from (select distinct g.name2, s.*
     from (select *, cast(chromEnd as signed integer)
                     -cast(chromStart as signed integer)
                     +cast(otherEnd as signed integer)
                     -cast(otherStart as signed integer) as length
          from genomicSuperDups
          where chrom=otherChrom and otherStart+1 between chromStart and chromEnd+101
          and fracMatch>=0.96 and strand="+" and
          cast(chromEnd as signed integer)
          -cast(chromStart as signed integer)
          +cast(otherEnd as signed integer)
          -cast(otherStart as signed integer) > 2000) s,
          wgEncodeGencodeBasicV17 g, wgEncodeGencodeAttrsV17 a
     where g.chrom=s.chrom and
           (((g.txStart<s.chromStart and g.txEnd>s.chromStart) and
           (g.txStart<s.chromEnd and g.txEnd>s.chromEnd)) or
           ((g.txStart<s.otherStart and g.txEnd>s.otherStart) and
           (g.txStart<s.otherEnd and g.txEnd>s.otherEnd)))
           and a.transcriptId=g.name and a.transcriptType='protein_coding') p,
     dgvMerged d
where d.pubMedId="23290073" and d.chrom=p.chrom and
      d.chromStart>p.chromStart and d.chromEnd<p.otherEnd
group by p.name2, p.chromStart, p.chromEnd, p.length
order by length desc;
```

Query 11 Query used to identify external structural variation at long tandem repeat loci (Table 3) using the data from Wong et al. [S39].

```
select p.name2, p.chromStart, p.chromEnd, length, sum(sampleSize) as samples,
       sum(observedGains) as gains, sum(observedLosses) as losses, count(*) as cnt
from (select distinct g.name2, s.*
     from (select *, cast(chromEnd as signed integer)
                     -cast(chromStart as signed integer)
                     +cast(otherEnd as signed integer)
                     -cast(otherStart as signed integer) as length
          from genomicSuperDups
          where chrom=otherChrom and otherStart+1 between chromStart and chromEnd+101
          and fracMatch>=0.96 and strand="+" and
          cast(chromEnd as signed integer)
          -cast(chromStart as signed integer)
          +cast(otherEnd as signed integer)
          -cast(otherStart as signed integer) > 2000) s,
          wgEncodeGencodeBasicV17 g, wgEncodeGencodeAttrsV17 a
     where g.chrom=s.chrom and
           (((g.txStart<s.chromStart and g.txEnd>s.chromStart) and
           (g.txStart<s.chromEnd and g.txEnd>s.chromEnd)) or
           ((g.txStart<s.otherStart and g.txEnd>s.otherStart) and
           (g.txStart<s.otherEnd and g.txEnd>s.otherEnd)))
           and a.transcriptId=g.name and a.transcriptType='protein_coding') p,
     dgvMerged d
where d.pubMedId="23290073" and d.chrom=p.chrom and
      d.chromStart<p.chromStart and d.chromEnd>p.otherEnd
group by p.name2, p.chromStart, p.chromEnd, p.length
order by length desc;
```

Query 12 Query used to identify internal structural variation at Self-chain loci (Table 4) using the data from Wong et al. [S39].

```
select p.name2, p.length, p.length2, matchlen, sum(sampleSize) as samples,
       sum(observedGains) as gains, sum(observedLosses) as losses, count(*) as cnt
from (select distinct g.name2, g.chrom, s.*
     from (select *, tEnd-tStart as length, qEnd-qStart as length2,
                  score/normscore as matchLen
          from chainSelf
          where tEnd-tStart>=5000 and qstrand="+" and normscore >=60 and tName=qName) s,
          wgEncodeGencodeBasicV17 g, wgEncodeGencodeAttrsV17 a
     where g.chrom=s.tName and g.txStart<s.tStart and g.txEnd> s.tEnd
           and g.txStart<s.qStart and g.txEnd> s.qEnd
           and a.transcriptId=g.name and a.transcriptType='protein_coding') p,
     dgvMerged d
where d.pubMedId="23290073" and d.chrom=p.chrom and
      ((d.chromStart>p.tStart or d.chromStart>p.qStart)
      and (d.chromEnd<p.tEnd or d.chromEnd<p.qEnd ))
group by p.name2, p.length, p.length2, matchlen
order by matchlen desc;
```

References

[S1] Bacolla A, Wells RD (2004) Non-B DNA conformations, genomic rearrangements, and human disease. J Biol Chem 279: 47411–47414.

[S2] Lobachev KS, Rattray A, Narayanan V (2007) Hairpin- and cruciform-mediated chromosome breakage: causes and consequences in eukaryotic cells. Front Biosci 12: 4208–4220.

[S3] Klein R, Jaenichen R, Zachau HG (1993) Expressed human immunoglobulin kappa genes and their hypermutation. Eur J Immunol 23: 3248–3262.

[S4] Rosenfeld MR, Wong E, Dalmau J, Manley G, Posner JB, et al. (1993) Cloning and characterization of a Lambert-Eaton myasthenic syndrome antigen. Ann Neurol 33: 113–120.

[S5] Smoller JW, Craddock N, Kendler K, Lee PH, Neale BM, et al. (2013) Identification of risk loci with shared effects on five major psychiatric disorders: a genome-wide analysis. Lancet 381: 1371–1379.

[S6] Toba S, Tenno M, Konishi M, Mikami T, Itoh N, et al. (2000) Brain-specific expression of a novel human UDP-GalNAc:polypeptide N-acetylgalactosaminyltransferase (GalNAc-T9). Biochim Biophys Acta 1493: 264–268.

[S7] Iwasaki H, Zhang Y, Tachibana K, Gotoh M, Kikuchi N, et al. (2003) Initiation of O-glycan synthesis in IgA1 hinge region is determined by a single enzyme, UDP-N-acetyl-alpha-D-galactosamine:polypeptide N-acetylgalactosaminyltransferase 2. J Biol Chem 278: 5613–5621.

[S8] Zamze S, Harvey DJ, Pesheva P, Mattu TS, Schachner M, et al. (1999) Glycosylation of a CNS-specific extracellular matrix glycoprotein, tenascin-R, is dominated by O-linked sialylated glycans and "brain-type" neutral N-glycans. Glycobiology 9: 823–831.

[S9] Yang H, Xiao ZC, Becker B, Hillenbrand R, Rougon G, et al. (1999) Role for myelin-associated glycoprotein as a functional tenascin-R receptor. J Neurosci Res 55: 687–701.

[S10] Bartsch U, Pesheva P, Raff M, Schachner M (1993) Expression of janusin (J1-160/180) in the retina and optic nerve of the developing and adult mouse. Glia 9: 57–69.

[S11] Pahlsson P, Spitalnik SL (1996) The role of glycosylation in synthesis and secretion of beta-amyloid precursor protein by Chinese hamster ovary cells. Arch Biochem Biophys 331: 177–186.

[S12] Tomita S, Kirino Y, Suzuki T (1998) Cleavage of Alzheimer's amyloid precursor protein (APP) by secretases occurs after O-glycosylation of APP in the protein secretory pathway. Identification of intracellular compartments in which APP cleavage occurs without using toxic agents that interfere with protein metabolism. J Biol Chem 273: 6277–6284.

[S13] Kitazume S, Tachida Y, Kato M, Yamaguchi Y, Honda T, et al. (2010) Brain endothelial cells produce amyloid beta from amyloid precursor protein 770 and preferentially secrete the O-glycosylated form. J Biol Chem 285: 40097–40103.

[S14] Oellerich T, Bremes V, Neumann K, Bohnenberger H, Dittmann K, et al. (2011) The B-cell antigen receptor signals through a preformed transducer module of SLP65 and CIN85. EMBO J 30: 3620–3634.

[S15] Hoffmann A, Grimm C, Kraft R, Goldbaum O, Wrede A, et al. (2010) TRPM3 is expressed in sphingosine-responsive myelinating oligodendrocytes. J Neurochem 114: 654–665.

[S16] Soliven B (2001) Calcium signalling in cells of oligodendroglial lineage. Microsc Res Tech 52: 672–679.

[S17] Fallarino F, Volpi C, Fazio F, Notartomaso S, Vacca C, et al. (2010) Metabotropic glutamate receptor-4 modulates adaptive immunity and restrains neuroinflammation. Nat Med 16: 897–902.

[S18] Frank M, Schaeren-Wiemers N, Schneider R, Schwab ME (1999) Developmental expression pattern of the myelin proteolipid MAL indicates different functions of MAL for immature Schwann cells and in a late step of CNS myelinogenesis. J Neurochem 73: 587–597.

[S19] Wright C, Sibani S, Trudgian D, Fischer R, Kessler B, et al. (2012) Detection of multiple autoantibodies in patients with ankylosing spondylitis using nucleic acid programmable protein arrays. Mol Cell Proteomics 11: M9.00384.

[S20] Zou J, Zhang Y, Thiel A, Rudwaleit M, Shi SL, et al. (2003) Predominant cellular immune response to the cartilage autoantigenic G1 aggrecan in ankylosing spondylitis and rheumatoid arthritis. Rheumatology (Oxford) 42: 846–855.

[S21] Kawaguchi Y, Osada R, Kanamori M, Ishihara H, Ohmori K, et al. (1999) Association between an aggrecan gene polymorphism and lumbar disc degeneration. Spine 24: 2456–2460.

[S22] Koutsilieri E, Lutz MB, Scheller C (2013) Autoimmunity, dendritic cells and relevance for Parkinson's disease. J Neural Transm 120: 75–81.

[S23] Monahan AJ, Warren M, Carvey PM (2008) Neuroinflammation and peripheral immune infiltration in Parkinson's disease: an autoimmune hypothesis. Cell Transplant 17: 363–372.

[S24] Nolan YM, Sullivan AM, Toulouse A (2013) Parkinson's disease in the nuclear age of neuroinflammation. Trends Mol Med 19: 187–196.

[S25] Sen N, Hara MR, Ahmad AS, Cascio MB, Kamiya A, et al. (2009) GOSPEL: a neuroprotective protein that binds to GAPDH upon S-nitrosylation. Neuron 63: 81–91.

[S26] Hara MR, Thomas B, Cascio MB, Bae BI, Hester LD, et al. (2006) Neuroprotection by pharmacologic blockade of the GAPDH death cascade. Proc Natl Acad Sci USA 103: 3887–3889.

[S27] Houlden H, Singleton AB (2012) The genetics and neuropathology of Parkinson's disease. Acta Neuropathol 124: 325–338.

[S28] Wang L, Nuytemans K, Bademci G, Jauregui C, Martin ER, et al. (2013) High-resolution survey in familial Parkinson disease genes reveals multiple independent copy number variation events in PARK2. Hum Mutat 34: 1071–1074.

[S29] Tan EK, Shen H, Tan JM, Lim KL, Fook-Chong S, et al. (2005) Differential expression of splice variant and wild-type parkin in sporadic Parkinson's disease. Neurogenetics 6: 179–184.

[S30] Chung JY, Park HR, Lee SJ, Lee SH, Kim JS, et al. (2013) Elevated TRAF2/6 expression in Parkinson's disease is caused by the loss of Parkin E3 ligase activity. Lab Invest 93: 663–676.

[S31] Northcott PA, Shih DJ, Peacock J, Garzia L, Morrissy AS, et al. (2012) Subgroup-specific structural variation across 1,000 medulloblastoma genomes. Nature 488: 49–56.

[S32] Pagani MR, Gonzalez LE, Uchitel OD (2011) Autoimmunity in amyotrophic lateral sclerosis: past and present. Neurol Res Int 2011: 497080.

[S33] Fiala M, Chattopadhay M, La Cava A, Tse E, Liu G, et al. (2010) IL-17A is increased in the serum and in spinal cord CD8 and mast cells of ALS patients. J Neuroinflammation 7: 76.

[S34] Hemminki K, Li X, Sundquist J, Sundquist K (2009) Familial risks for amyotrophic lateral sclerosis and autoimmune diseases. Neurogenetics 10: 111–116.

[S35] Bonifacino JS, Hierro A (2011) Transport according to GARP: receiving retrograde cargo at the trans-Golgi network. Trends Cell Biol 21: 159–167.

[S36] Perez-Victoria FJ, Abascal-Palacios G, Tascon I, Kajava A, Magadan JG, et al. (2010) Structural basis for the wobbler mouse neurodegenerative disorder caused by mutation in the Vps54 subunit of the GARP complex. Proc Natl Acad Sci USA 107: 12860–12865.

[S37] Naslund K, Saetre P, von Salome J, Bergstrom TF, Jareborg N, et al. (2005) Genome-wide prediction of human VNTRs. Genomics 85: 24–35.

[S38] Legendre M, Pochet N, Pak T, Verstrepen KJ (2007) Sequence-based estimation of minisatellite and microsatellite repeat variability. Genome Res 17: 1787–1796.

[S39] Wong LP, Ong RT, Poh WT, Liu X, Chen P, et al. (2013) Deep whole-genome sequencing of 100 southeast Asian Malays. Am J Hum Genet 92: 52–66.

[S40] Taggart RT, Samloff IM, Raffel LJ, Graham A, Cass C, et al. (1986) Relationships between the human pepsinogen DNA and protein polymorphisms. Am J Hum Genet 38: 848–854.

[S41] Conrad DF, Pinto D, Redon R, Feuk L, Gokcumen O, et al. (2010) Origins and functional impact of copy number variation in the human genome. Nature 464: 704–712.

[S42] Kidd JM, Cooper GM, Donahue WF, Hayden HS, Sampas N, et al. (2008) Mapping and sequencing of structural variation from eight human genomes. Nature 453: 56–64.

[S43] Pruitt KD, Tatusova T, Brown GR, Maglott DR (2012) NCBI Reference Sequences (RefSeq): current status, new features and genome annotation policy. Nucleic Acids Res 40: D130–135.
